# Waste‐Contaminated Sites Near Dhaka City Harbour Specific Biosurfactant‐Producing *Bacillus* and *Stutzerimonas* Strains With Potential Biotechnological Applications

**DOI:** 10.1111/1758-2229.70440

**Published:** 2026-10-23

**Authors:** Ummey Fatema Tun Amina, Maliha Mahzabin, Sk. Shahriar Bin Rasul, Sabrina M. Elias

**Affiliations:** ^1^ Department of Life Sciences, School of Environment and Life Sciences Independent University Bangladesh Dhaka Bangladesh; ^2^ IUB Plasma Plus Research and Testing Laboratory, School of Pharmacy and Public Health Independent University Bangladesh Dhaka Bangladesh; ^3^ The Center for Computational and Data Sciences Independent University Bangladesh Dhaka Bangladesh

**Keywords:** bacteria, bioremediation, biosurfactant, waste

## Abstract

Biosurfactants are eco‐friendly surface‐active compounds produced by microorganisms that lower surface and interfacial tensions, enhancing the bioavailability of hydrophobic compounds. The study reports the isolation and characterization of biosurfactant‐producing bacteria from industrial waste effluent in a river near Dhaka, Bangladesh. Using diesel‐enriched media, bacterial strains were isolated and screened for biosurfactant production using oil displacement, emulsification index (E24%), and drop‐collapse assays. The microbial cultures were characterized based on biochemical activities and 16S rRNA sequencing. Biosurfactant characterization was carried out using chromatographic techniques. Six cultures passed the drop‐collapse and oil‐displacement tests with E24% ranging from 9% to 41%. The CTAB agar assay confirmed the anionic nature of the biosurfactants. The potential biosurfactant producers belong mostly to the *Bacillus* and *Stutzerimonas* groups, with one low‐biosurfactant‐producing isolate from the *Enterobacteriaceae* group. The presence of two consortia, *Bacillus‐Acinetobacter* and *Bacillus‐Stutzerimonas*, was also observed. The isolates and consortia exhibited varying levels of antibacterial, antifungal, adherence, and plant growth‐promoting activity. Biosurfactant production capacity under extreme conditions has been characterized by pH stability (pH 2–pH 11), salinity tolerance (2%–10% NaCl), and temperature tolerance (40°C–100°C). Overall, they provide a green and economical alternative to synthetic surfactants, with low toxicity and potential applications in bioremediation, biocontrol, and plant growth promotion.

## Graphical abstract

Biosurfactant‐producing bacteria were isolated from hydrocarbon‐contaminated industrial effluent in Dhaka, Bangladesh, using selective diesel liquid enrichment. Functional screening confirmed surfactant activity via oil displacement, drop‐collapse, and emulsification index assays. CTAB agar testing revealed anionic biosurfactants, while TLC and FTIR identified lipopeptides and glycolipids that maintained emulsion stability across extreme pH (2–12), temperature (40°C–100°C), and salinity (2%–10% NaCl). Isolates demonstrated effective hydrocarbon bioremediation, broad antibacterial and antifungal inhibition, and enhanced rice seedling growth under salt stress.

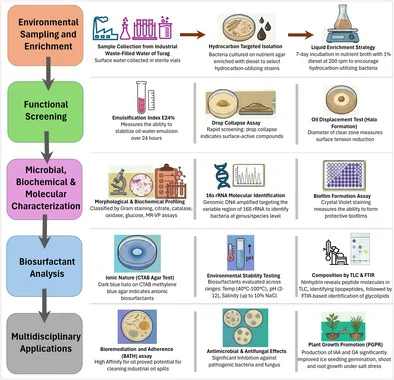

## 1 Introduction

Industrial waste typically consists of hydrocarbon waste, heavy metals, and other pollutants. Due to their hydrophobic nature, pollutants are difficult to degrade and pose a health risk to humans and the environment. Synthetic surfactants are widely used to manage pollutants because they are amphiphilic, enabling them to interact with both hydrophobic and hydrophilic compounds. Hence, surfactants aid in waste removal by emulsifying hydrophobic pollutants, enhancing solubility, and facilitating their breakdown or removal from contaminated environments. However, synthetic surfactants are often toxic, non‐renewable, and environmentally persistent, raising concerns about their long‐term sustainability (Banat et al. 2010).

Biosurfactants represent an eco‐friendly alternative due to their biodegradability and low toxicity. These compounds are produced by diverse microorganisms, including fungi, bacteria, and actinomycetes. Primarily located on microbial surfaces or sometimes secreted extracellularly, they can effectively reduce surface and interfacial tensions, enhance emulsification, and degrade hydrophobic pollutants. Secretion of these compounds helps the bacteria grow, spread, access hydrophobic nutrients, and persist in challenging environments. These compounds also provide them with advantages by disrupting competing microbes and improving root colonization. However, the biosurfactants produced by microbes are not only essential for their physiology and survival but also hold significant utility for the bioremediation of locations contaminated with hydrocarbons, heavy metals, and pesticides (Datta and Chattopadhyay 2024). Biosurfactants have been reported for use in a wide range of industrial domains, including environmental protection, agriculture, petroleum, food, and the pharmaceutical industry (Rostás and Blassmann 2009; Deleu and Paquot 2004; Singh et al. 2007). They serve as a biocontrol agent to shield plants against a variety of diseases, increasing agricultural production (Moldes et al. 2011; Simpson et al. 2011). According to Mulligan (2005), a high‐quality surfactant may reduce the interfacial tension between hexadecane and water from 40 to 1 mN/M and the surface tension of water from 75 to 35 mN/m (Muthusamy et al. 2008) (Makkar and Cameotra 1997). Temperatures as high as 50°C, a pH range of 4.0 to 9.0, a 50 g/L concentration of NaCl, and a 25 g/L concentration of Calcium did not impact the lichenysin produced by *Bacillus licheniformis* (Muthusamy et al. 2008). Biosurfactants are superior to chemical surfactants in terms of biocompatibility (Mohan et al. 2006), low toxicity (Desai and Banat 1997), biodegradability, accessibility of the basic materials, and stability at extreme pH and temperature (Muthusamy et al. 2008). Also, they often show anti‐bacterial, antifungal, and anti‐adhesive properties, which are beneficial for different therapeutic applications (Gudiña et al. 2010). Because of their versatile properties, the study of biosurfactants and the search for biosurfactant‐producing micro‐organisms remain essential and intriguing fields of research.

The most extensively studied biosurfactant producers belong to the bacterial genera *Pseudomonas, Bacillus*, and *Rhodococcus*, as well as the yeast *S. bombicola*. To date, over 2000 different structures of biosurfactants and their congeners have been identified (Dagenais Roy and Déziel 2025). Based on chemical structure, major biosurfactants are classified as phospholipids, glycolipids, lipopeptides, lipopolysaccharides, polymeric biosurfactants (bioemulsifiers), fatty acids, and neutral lipids. *Bacillus* spp. produces lipopeptides such as surfactin, fengycin, and iturin, which have surface‐active and antimicrobial properties (Saiyam et al. 2024). Surfactin is synthesized through non‐ribosomal biosynthesis, catalysed by surfactin synthetase. Other lipopeptides like iturin, lichenysin, and arthrofactin are synthesized by similar enzyme complexes. Among the glycolipids, rhamnolipids, sophorolipids, and trehalolipids are the most well‐known types (Md 2012). *Pseudomonades* are notable for producing rhamnolipids, which are widely used in bioremediation for their emulsification efficiency and hydrocarbon‐degrading ability. In *Pseudomonas* species, the *rhl* quorum‐sensing system is essential for glycolipid biosurfactant production. Biosurfactant composition and emulsifying activity are dependent on both the producer strain and the culture conditions; consequently, the type of carbon and nitrogen source, as well as the C:N ratio, nutritional limitations, chemical and physical parameters like pH, aeration, temperature, and divalent cations affect both the quantity of the manufactured biosurfactant as well as the kind of polymer produced (Salihu et al. 2009).

Urban waste disposal sites, industrial effluents, and oil‐contaminated soils are rich in microbial diversity adapted to survive in pollutant‐contaminated habitats and can be excellent sources of biosurfactant‐producing bacteria. Tongi, near the Dhaka metropolitan area, is one of the most industrialized and polluted regions in Bangladesh (Rabbani and Sarker 2017) that generates large amounts of industrial waste, stemming primarily from untreated industrial effluents such as textile, dyeing, and pharmaceutical waste, as well as municipal sewage outfalls. The heterogeneous nature of waste in this waterbody creates favourable conditions for harbouring biosurfactant‐producing bacteria naturally (Carolin C et al. 2023). Microbes adapting to such hydrocarbon‐contaminated environments evolve complex survival mechanisms. We hypothesized that their biosurfactant production serves as a dual‐action survival strategy, using hydrophobic substrates for bioremediation while simultaneously facilitating root colonization and antagonizing microbial competitors under environmental stress. The underexplored microbial diversity and their adaptation mechanisms need attention for systematic exploration, potential scalability for industrial applications, and the development of eco‐friendly, multidimensional uses in our daily lives.

The current study aims to explore and characterize biosurfactant‐producing bacteria from the industrial waste‐contaminated water sample from Turag Khal in Tongi. In this quest for biosurfactant‐producing bacteria, six potential cultures have been identified from this underexplored water hub. The workflow of the study is depicted in Figure 1. Two of them were found to be mixed cultures. These isolates and consortia were screened for biosurfactant production using the oil displacement test, emulsification index (E24%), and drop‐collapse assay. The potential ones were further investigated for their metabolic activities and genetic identity, as well as their biosurfactant‐producing ability under extreme pH, temperature, and salinity stress. Finally, the antibacterial, antifungal, and plant growth‐promoting properties of these surfactants, along with the bacterial isolates, were assessed for viability as an alternative to synthetic surfactants. Based on their diverse properties, the isolates were categorized for potential applications upon future scale‐up of biosurfactant production.

**FIGURE 1:**
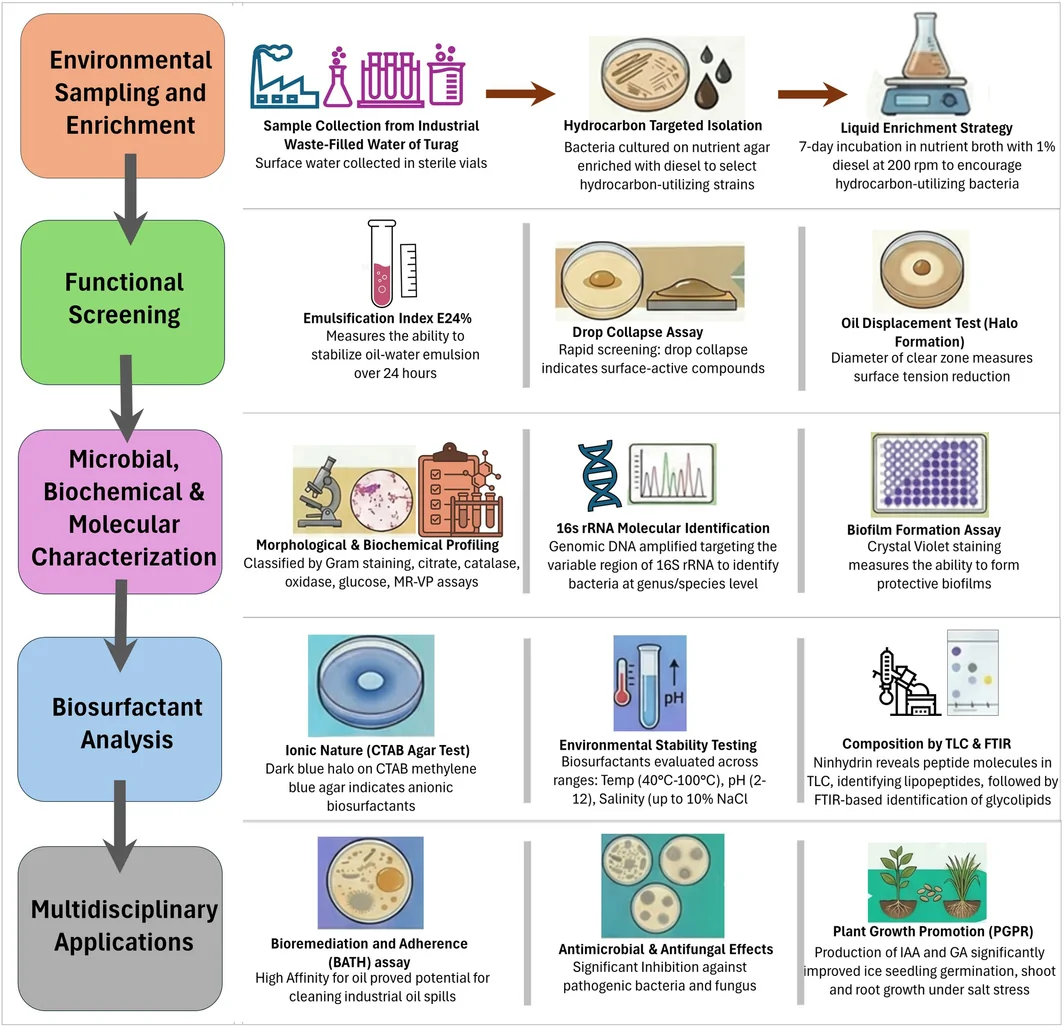
Schematic diagram of the workflow.

## 2 Experimental Procedures

### 2.1 Sample Collection and Site Characterization

Water samples were collected from the waste effluent water body, Tongi Khal, near the industrial zone of Tongi, a city close to the capital city, Dhaka, Bangladesh. The sampling site was located at the wastewater discharge point, contaminated with untreated industrial effluents and municipal sewage, at approximately 23.8924° N, 90.3599° E. The samples were collected in sterile vials, sent immediately to the laboratory at Independent University, Bangladesh, and were stored there at 4°C pending further examination. The presence of hydrocarbon contamination in the water was tested using UV light and compared with a pond water sample not contaminated with industrial waste, based on the principle that polycyclic aromatic hydrocarbons (PAHs) naturally absorb UV light and re‐emit it as a greenish colour. Alkalinity, hardness, pH, and heavy metal contamination in the water sample were assessed using a strip‐based water testing kit (16 in 1 Water Test Strips) and the Aquasol AE408 Arsenic testing kit. The water testing strips assessed pH, total alkalinity, carbonate, hardness, cyanuric acid, copper, mercury, total chlorine, free chlorine, bromine, nitrite, nitrate, iron, chromium (V1), lead and Fluoride.

### 2.2 Isolation and Enrichment Method

Nutrient broth and agar medium were used to isolate biosurfactant‐producing bacteria. The spread plate method was used to inoculate the six serially diluted samples on nutrient agar. The samples were incubated for 24 h at 37°C in an aerobic environment. Each Petri dish contained diesel as the hydrocarbon source, and a control without diesel was additionally maintained. Diesel was added to the solid media on the plate using cotton buds for enrichment. Morphologically distinct colonies were sub‐cultured onto another agar plate to obtain a pure culture of the organism.

### 2.3 Inoculum Preparation

From the pure culture obtained, twenty‐four‐hour‐old colonies were inoculated into 100 mL of nutrient broth containing 1% (v/v) diesel and incubated at 30°C with shaking at 200 rpm for 7 days (Dunlap and Beckmann 1988). From this one‐week incubated vial, 1 mL of the culture was transferred to 99 mL of nutrient broth containing 1% diesel and incubated for 2 days at 30°C with shaking at 200 rpm. After 2 days, 1 mL of the culture from this vial was transferred to fresh 99 mL of nutrient broth containing 1% diesel, and the sample was incubated for 1 day, bringing the total preparation time to 10 days.

### 2.4 Screening of the Isolates for Biosurfactant Production

Biosurfactants usually show extracellular production; hence, the cell‐free extract was obtained by centrifugation and utilized for subsequent examination (Femi‐Ola et al. 2015). The culture medium was centrifuged at 3000 rpm for 30 min; the cell pellet was discarded, and the supernatant was collected for use in subsequent screening experiments. Additionally, the cell‐free extract was acid‐precipitated and semi‐purified using chloroform and methanol. The screening tests described below were performed using the cell‐free extract. Additionally, oil displacement and emulsification tests were repeated with the semi‐purified compounds.

#### 2.4.1 Oil Displacement Test

The biosurfactant's effectiveness in reducing surface tension was determined by measuring the diameter of the clear zone that appeared after adding a biosurfactant‐containing solution to an oil–water interface (Morikawa et al. 2000). A 90 mm Petri dish was filled with 15 mL of distilled water, then 100 μL of diesel was added to the water surface, forming a thin oil layer. Subsequently, 20 μL of cell‐free supernatant was gently dispensed onto the center of the oil layer. After 30 s, the circumference and the visible oil‐displacement halo were measured (Figure 2a). A drop of water served as the negative control. The same steps were performed using 20 μL chloroform‐methanol extracts instead of cell‐free supernatants, termed as semi‐purified biosurfactant.

**FIGURE 2:**
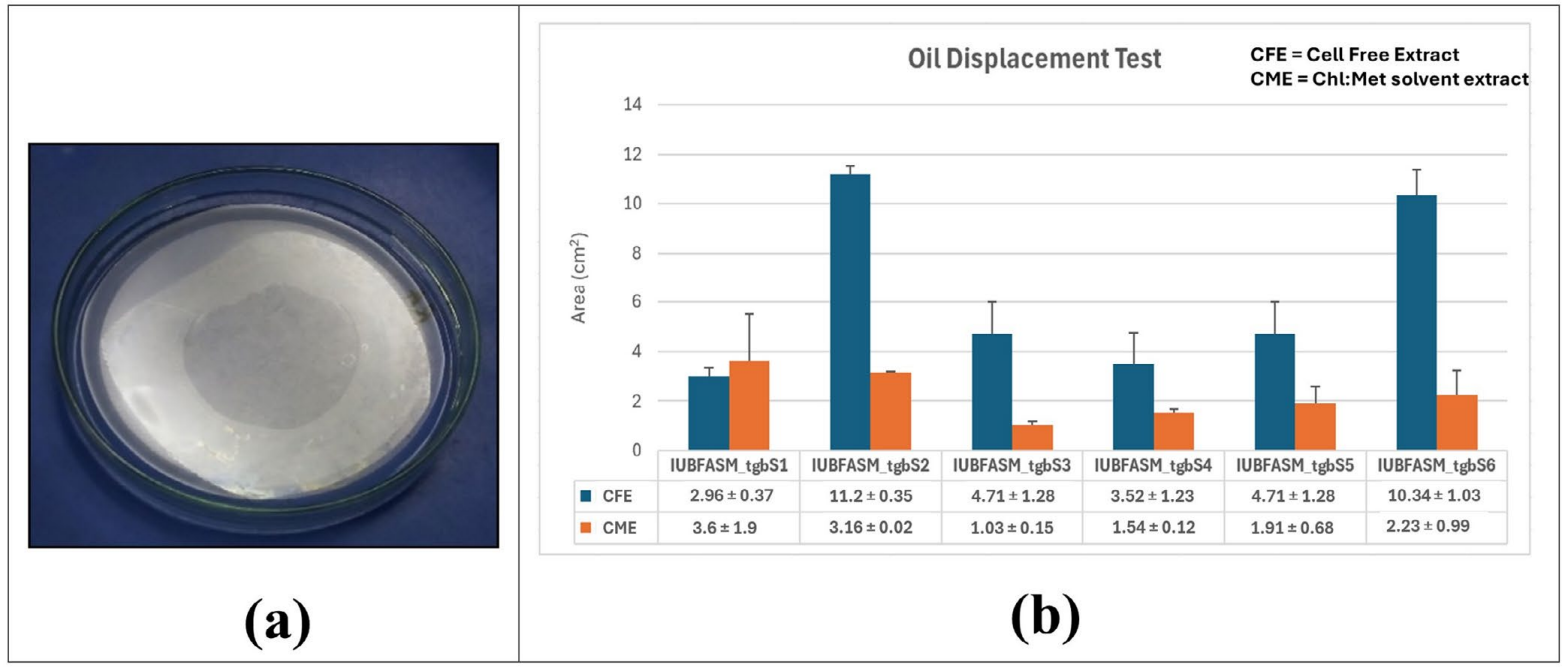
(a) Oil displacement test showing the presence of a clear zone or halo in IUBFASM_tgbS6 (b) Oil Displacement ability of the six isolates in terms of area (cm^2^), comparing both CFE (Cell Free Extract) and semi‐purified CME (Chloroform: Methanol Extract).

#### 2.4.2 Emulsification Index (E24%) Measurement

The Cooper and Goldenberg method was used to determine the biosurfactant's emulsification capacity towards diesel and kerosene (Cooper and Goldenberg 1987). In a test tube, 1 mL of cell‐free extract or semi‐purified surfactant (in a separate experiment) and 2 mL of oil were combined. The mixture was vortexed for 2 min and was allowed to stand undisturbed at room temperature for 24 h (Figure 3a,b). After incubation, the emulsion activity was assessed by measuring the heights of the stable emulsion layer and the total liquid layer. The emulsification index was calculated using Equation (1).

(1) $$E24\left(\%\right)=\frac{\text{Height of the emulsified layer}}{\text{Total height of the liquid column}}\times 100$$

**FIGURE 3:**
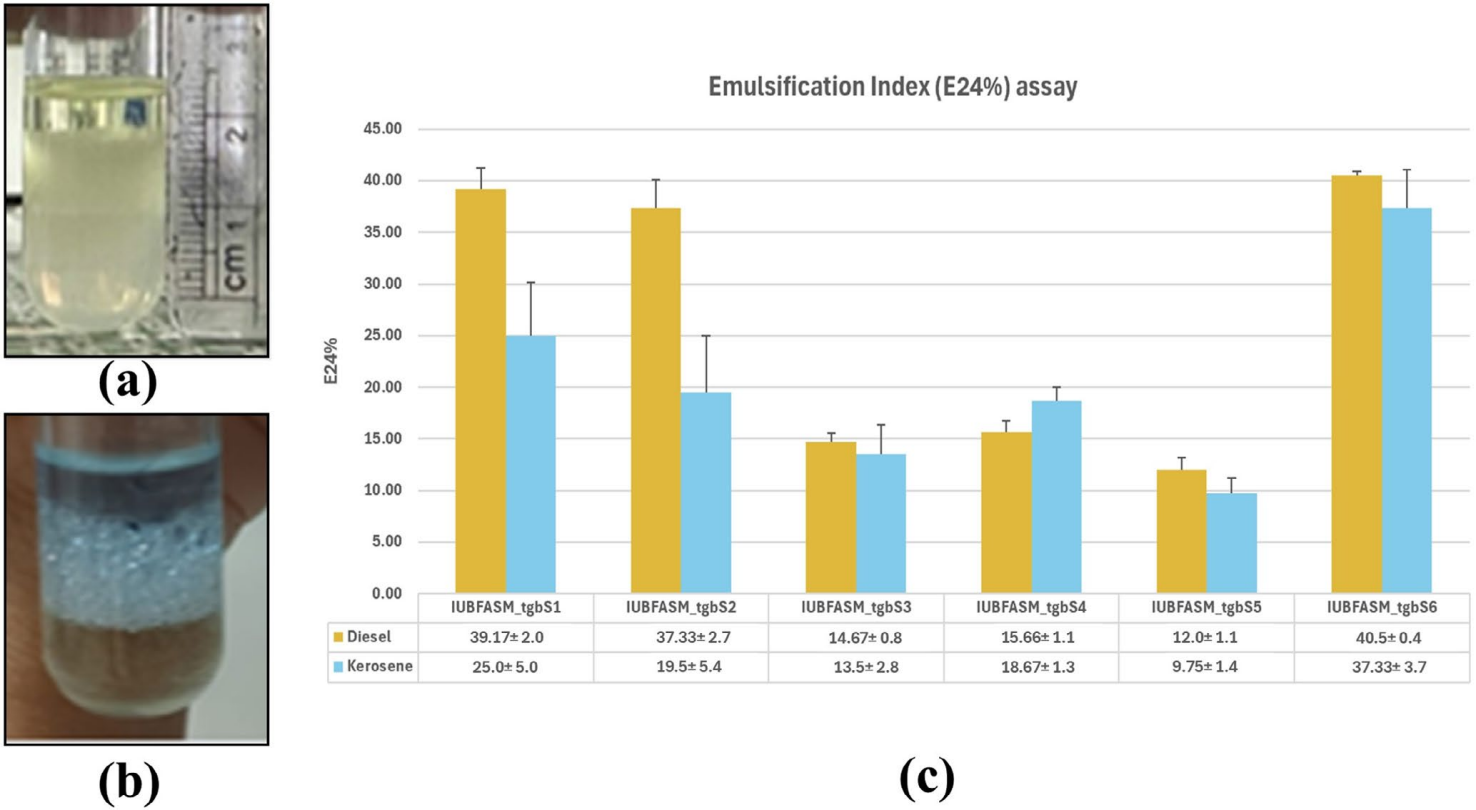
Emulsification Assay: Emulsification Assay of IUBFASM_tgbS6 in Diesel (a) and Kerosene (b). Emulsification Index of all isolates using cell‐free extracts (c).

All measurements were replicated 3 times, and the results were expressed as the mean ± standard error. As a positive control, 10% Sodium Dodecyl Sulfate was used.

#### 2.4.3 Drop Collapse Assay

The experiment was conducted following instructions provided by Jain et al. (1991). In a 96‐well microtiter plate lid, each well was filled with 2 μL of oil and left for 1 h at room temperature. After this, 5 μL of the bacterial culture supernatant was added to the oil surface. The drop shape was visually inspected after 1 min. Production of flattened, collapsed drops was considered positive for biosurfactant production, indicating surface activity. On the other hand, stable, round drops indicated the absence of biosurfactant activity. Distilled water was used as a negative control in the experiment.

### 2.5 Biochemical and Molecular Characterization

#### 2.5.1 Gram Staining

Gram staining was performed to differentiate Gram‐positive and Gram‐negative bacteria based on the thickness of their peptidoglycan layer. A bacterial smear was heat‐fixed on a slide, stained with crystal violet, then treated with iodine, decolorized with ethanol, and counterstained with safranin. The results were observed under a light microscope. In addition to staining, a loopful of bacteria was mixed with 3% KOH for 60 s, and the appearance of a sticky, string‐like structure was observed in gram‐negative cultures, while the absence of such a structure confirmed the gram‐positive nature of the culture.

#### 2.5.2 Biochemical Tests for Metabolic and Enzymatic Characterization

A series of biochemical tests was carried out following standard microbiological procedures to determine the metabolic and enzymatic activities of the isolates.

##### 2.5.2.1 Motility, Indole and Urease (MIU) Test

The motility, indole production, and urease activity of the bacterial isolates were determined using MIU medium. Each isolate was inoculated in the MIU medium by a single stab with a sterile needle and incubated at 37°C for 24 h. Motility was confirmed by observing bacteria growing away from the stab line, forming turbidity. Urease activity was confirmed by observing a pink‐red colour, and indole production was determined by the formation of a red ring at the surface after the addition of Kovac's reagent.

##### 2.5.2.2 Kligler Iron Agar (KIA) Test

In KIA agar, the bacterial culture was inoculated by stabbing followed by streaking and was incubated at 37°C for 24 h. Representative colour changes and the presence of gas or H_2_S were observed.

##### 2.5.2.3 Citrate Utilization Test

Bacterial Isolates were streaked onto Simmons' citrate agar slants and incubated at 37°C for 24 h. Positive citrate utilization was indicated by a colour change from green to blue, which confirms their ability to use citrate as a sole carbon source.

##### 2.5.2.4 Starch Hydrolysis Test

Bacterial cultures were streaked onto starch agar plates and incubated at 37°C for 24 h, then flooded with iodine solution. A clear zone around the bacterial growth indicated positive starch hydrolysis, confirming amylase production, whereas the absence of a clear zone with blue‐black coloration indicated negative starch hydrolysis.

##### 2.5.2.5 Oxidase Test

A fresh bacterial colony was smeared on a filter paper strip soaked with N,N,N′, N′‐Tetramethyl‐1,4‐phenylenediamine. A positive oxidase test was indicated by a colour change to dark purple within 30 s, suggesting the presence of cytochrome c oxidase.

##### 2.5.2.6 Catalase Test

A loopful of respective bacterial culture was taken on a glass slide, and 3% hydrogen peroxide was added as a drop. The presence of a gas bubble indicates a positive catalase reaction.

##### 2.5.2.7 Methyl Red (MR) and Voges‐Proskauer (VP) Test

Bacterial culture was inoculated into MR‐VP broth and incubated for 48 h at 37°C. For the MR test, methyl red indicator was added in 5 drops. A red colour indicates a positive MR test, meaning stable acid has been produced from glucose fermentation. For the VP test, Barritt's A (α‐naphthol) and Barritt's B (KOH) solutions were added to the incubated bacterial culture in MR‐VP broth. Development of a reddish colour within 15–30 min indicated a positive VP test, suggesting acetoin production.

#### 2.5.3 16S rRNA‐Based Squencing

To identify the isolate at the molecular level, 16S rRNA gene analysis was performed. With the use of a ProFlex PCR system, the 16S rRNA gene was amplified from the genomic DNA using the universal forward primer 27F (5′‐AGTTTGATCCTGGCTCAG‐3′) and reverse primer 1492 R (5′‐AAGGAGGTGATCCAGCC‐3′). The PCR reaction consisted of ~50 ng of DNA template, 1X PCR buffer, 1.6 mM MgCl_2_, 0.1 mM dNTPs, and 0.33 μM of forward and reverse primers adjusted with nuclease‐free water. In the PCR protocol, denaturation was carried out for 5 min at 95°C, annealing for 1 min at 57°C, and extension for 7 min at 72°C. This was followed by thirty‐five cycles of temperature cycling. The amplified 16S rRNA gene was isolated using electrophoresis on 0.8% (w/v) agarose gel followed by purification using the QIAgen gel extraction kit. Sanger sequencing was performed on the Genetic Analyser 3500 from Applied Biosystems (Thermo Fisher Scientific Inc.). The sequences were aligned with the NCBI 16S rRNA database, and the database entry yielding the highest similarity index was used to identify the unknown bacterium. However, for further confirmation of the culture, 16S rRNA amplicon‐based next‐generation sequencing was performed, targeting the hypervariable V3 and V4 region of the bacterial 16S rRNA using the universal primer as described in (Klindworth et al. 2012). The amplicon library was prepared on a Miseq Illumina platform with 2 × 300 bp paired‐end reads. QIIME 2 (version 2025.7) (Bolyen et al. 2019) was used for processing raw reads. Demultiplexed reads were quality assessed, and primer/adapter and low‐quality reads were trimmed as necessary using the built‐in features of the DADA2 plugin in QIIME 2, prior to downstream analyses. Amplicon Sequence Variants (ASVs) were inferred using DADA2 and the feature‐table option in QIIME 2. The representative ASVs were classified taxonomically using a naïve Bayes classifier trained on the SILVA reference database (release 138.1) trimmed to the V3–V4 region. Taxonomic assignments were accepted using the default confidence threshold (0.7) unless otherwise specified.

#### 2.5.4 Biofilm Formation Assay

A loopful of each bacterial isolate was inoculated into 5 mL of nutrient broth and incubated at 37°C overnight. Biofilm formation was evaluated in a U‐bottom 96‐well polystyrene microtiter plate. Each well of the plate was filled with 100 μL of the 100‐fold diluted bacterial culture. The plates were incubated at 37°C for 24 h under static conditions to allow biofilm development. Following incubation, the culture medium containing planktonic cells was carefully removed, and each well was gently rinsed three times with sterile distilled water to eliminate non‐adherent bacteria. The plates were then air‐dried at room temperature. Subsequently, 125 μL of 0.1% (w/v) crystal violet solution was added to each well and allowed to stain the attached biofilm for 10 min. Excess stain was discarded, and the wells were washed thoroughly with distilled water to remove unbound dye. The plates were again air‐dried to ensure complete fixation of the stained biomass (O'Toole 2011). In each well, 200 μL of ethanol was added, and after 15 min of mixing, the optical density (OD) at 600 nm was measured (Sharma and Kaur 2015). For the 10‐day post‐incubation biofilm formation assay, the same inoculum preparation steps were followed as stated in Section 2.3. Twenty‐four‐hour‐old colonies were cultured in 1% (v/v) diesel at 30°C and 200 rpm for 7 days and sequentially sub‐cultured twice (1% v/v inoculum in identical diesel‐enriched fresh media) for an additional 2 days and 1 day, totaling 10 days of preparation. This culture was then diluted and applied to the U‐bottom 96‐well polystyrene microtiter plate for biofilm evaluation following the steps as stated above.

### 2.6 Characterization of the Surfactant Compound

#### 2.6.1 Ionic Characterization via CTAB Agar Test

To detect anionic biosurfactants, the CTAB agar plate method was used. The medium was prepared by mixing 1 L of distilled water with 0.15 g of cetyltrimethylammonium bromide (CTAB), 0.005 g of methylene blue, and 12 g of agar to BHM (Bushnell‐Haas Mineral). The pH was adjusted to 7.0, and the medium was sterilized (Tahzibi et al. 2004). After solidification, two wells (6.5 mm in diameter) were aseptically made in each plate. About 100 μL of sterile‐filtered cell‐free supernatant was added to each well. The plates were incubated at 37°C for 48 h. The appearance of distinct blue halos around the wells indicated the production of anionic biosurfactants. Halo diameters were measured and compared to positive and negative controls.

#### 2.6.2 Emulsion Stability Studies

Emulsion stability tests were performed to determine the optimal conditions for the stability and functionality of biosurfactants produced by the isolates. The E24% experiment (using diesel) was conducted to determine emulsion stability across various temperatures, pH levels, and salt concentrations. By completing the E24% assay of cell‐free culture with diesel and incubating the resulting emulsion at 40°C, 80°C, and 100°C, the impact of temperature on emulsion stability was assessed. By adjusting the cell‐free broth's pH (using 1 N HCl or 1 N NaOH) or salt concentration (by adding NaCl) to the appropriate levels and computing E24% indices after 24 h, the impact of pH and salinity on emulsion stability was measured. Salt concentration ranged from 2% to 10% (2%, 6%, and 10%), whereas pH ranged from 2.0 to 12.0 (2.0, 7.0, and 12.0).

#### 2.6.3 ThinLayer Chromatography

The biosurfactant‐producing isolates were cultured in 100 mL of BHM (Bushnell‐Huss Mineral) broth supplemented with 2% (w/v) glucose, 2% (v/v) molasses, and 2% (v/v) oil. This culture was kept with agitation at 200 rpm at 30°C for 7 days. Cell‐free supernatants were generated by centrifugation at 5200 rpm at 4°C to remove bacterial cells. This cell‐free supernatant was then treated with 2 N HCl for 16 h at 4°C to reach a final pH of 2.0. The solution was then centrifuged at 5200 rpm for 10 min at 4°C, and the resulting precipitate was collected. This acid precipitate fraction (APF) was then dissolved in 100 mg/mL water, and the pH was adjusted to 7.0 using 1 N NaOH (Rani et al. 2020) The CFS sample and clean BHM culture medium were stored at −20°C until further use. Thin‐layer chromatography (TLC) was used to separate the CFS and APF fractions. Before use, the TLC plates were heated to 120°C for 30 min to activate the silica plates. Chloroform: methanol: water (70:26:4) was used as the developing solvent system for the separation of 100 mg each of CFS and APF in 400 μL of chloroform: methanol (2:1, v/v) on activated TLC plates. Plates were examined in UV light (λ = 254 nm), and the retention factor (Rf) values were computed by dividing the sample's travel distance by the solvent's travel distance (Equation 2). The dry plates were sprayed with a 0.25% (w/v) ninhydrin in acetone solution and incubated at 115°C for 5 min to detect peptides (Xia et al. 2014).

(2) $$\text{Retention Factor}\;\left(\mathrm{Rf}\right)=\frac{\text{Distance traveled}\;\mathrm{by}\;\text{the compound}}{\text{Distance traveled}\;\mathrm{by}\;\text{the solvent front}}$$

### 2.7 Additional Properties of the Bacterial Strains

#### 2.7.1 Bacterial Adhesion to Hydrocarbons (BATH) Assay

Cell viability in the presence of oil was examined using the BATH assay. After centrifugation of the overnight‐grown culture in broth, it was resuspended three times in phosphate buffer (16.9 g/L K_2_HPO4 and 7.3 g/L KH_2_PO4). At 610 nm, the optical density was approximately 0.5. After vortexing 2 mL of cell suspension with 100 μL of oil for 3 min, the aqueous layer was separated. The oil coating was removed, and the optical density was measured following 1 h of undisturbed incubation (Van Der Vegt et al. 1991). The following formula (Equation 3) was used to determine the % adherence to oil:

(3) $$\%\text{Adherence}=\frac{\left(\text{Initial}\;\mathrm{OD}-\text{Final}\;\mathrm{OD}\right)}{\text{Initial}\;\mathrm{OD}}\times 100$$

where initial OD refers to the optical density of the cell suspension in buffer solution before mixing with oil, and final OD (*also known as OD*_*Shaken*_) refers to the optical density of the aqueous phase after the cell suspension and the oil have been mixed and allowed to separate. Thus, it represents the bacterial cells remaining in the aqueous phase that did not adhere to the oil.

#### 2.7.2 Agar Diffusion Assay to Test Antimicrobial Activity

Using the Burkholder agar diffusion assay, the strains were further examined for their antibacterial efficacy against a broad panel of clinical and environmental bacterial species (Burkholder et al. 1944). To reach mid‐log phase and a cell density of 10^6^ CFU/mL, all target bacterial strains and experimental isolates were cultured in LB broth at 27°C for 16–18 h with constant shaking. After centrifugation at 5800 rpm for 20 min at 4°C, the cells were suspended in sterile deionized water. Each bacterial suspension (80 μL) was then combined with 7 mL of melted half‐strength LB agar, and the mixture was transferred into 15‐mm‐diameter culture plates. The plates were incubated at 24°C after an aliquot (15 μL) of experimental isolates was placed on the bacterial lawn. The plate was divided into 3 zones, and the isolates were placed in the middle of the zones. After 24 to 48 h, zones of bacterial growth inhibition were observed adjacent to the inoculum of the spotted isolates. The diameter of these zones varied based on the efficacy of the isolates' antimicrobials that permeated into the agar (Rani et al. 2020). We tested the antimicrobial activity of the experimental isolates against clinically infectious *Shigella, E coli, Ogawa, Klebsiella*, and *Staphylococcus aureus*. The same procedure was also performed with the semi‐purified biosurfactant extracts instead of the bacterial isolates.

#### 2.7.3 Antibacterial Susceptibility Assay

The Kirby‐Bauer test was performed to measure the isolates' susceptibility to known antibiotics and assess whether these bacteria would be safe for future use. A standardized bacterial suspension (matched to a McFarland 0.5 turbidity standard) was spread evenly across a Mueller‐Hinton agar plate. Commercial antibiotic discs for ampicillin, chloramphenicol, oxytetracycline, and azithromycin were used for checking susceptibility.

#### 2.7.4 Antifungal Activity

Antifungal activity of biosurfactant extracts was evaluated using the agar‐well diffusion method. Potato Dextrose Agar (PDA) was used as the medium on a plate, and four wells were created 3 cm from the plate center. At the center, a 0.3 cm diameter mycelial plug of the fungus was inoculated. In the control well, 50 μL of methanol was added, and in the remaining wells, 50 μL of the biosurfactant solutions was added. Here, we used two types of biosurfactant solutions. One is the cell‐free supernatant, and the other is its filtered version.

We also assessed the antifungal activity of the biosurfactant inoculum using the spread plate method. 3 mL of the biosurfactant inoculum was evenly spread onto the surface of each Potato Dextrose Agar (PDA) plate. A fungal mycelial plug (approximately 3 mm in diameter) was aseptically placed at the center of each plate. The plates were incubated at room temperature (25°C ± 2°C) for a specified period to assess fungal growth inhibition. Activity was assessed against plant‐pathogenic fungi, including *Colletotrichum* sp. and *Pestalotiopsis*, as well as various non‐pathogenic fungal species.

#### 2.7.5 Plant Growth Promotion Activity

##### 2.7.5.1 Rice Seedling Stage Screening Upon Application of Bacteria

Bacterial culture was prepared using overnight growth of the bacteria in Nutrient broth (NB) at 37°C. Using 1% (v/v) sodium hypochlorite, rice seeds were surface‐sterilized and washed 3 times with sterile water to remove residual chemicals. Then the seeds were treated with an overnight‐grown bacterial culture to observe the changes in germination and growth. We used two experimental groups: one in which the seeds were soaked in 2 mL of bacterial culture for 2 h with shaking, and the other in which the seeds were immersed in 2 mL of bacterial culture containing 150 mM NaCl. The seeds were then transferred to sterile Petri dishes containing 4 mL of sterile water, and the moisture level was maintained for 7 days. A similar experiment was carried out, submerging the seeds in 2 mL of cell‐free supernatants to observe the effects of the surfactant. Seed germination percentage was monitored daily during incubation at 28°C. The germination percentage was calculated as the number of germinated seeds divided by the total number of seeds, multiplied by 100 (Equation 4).

Seedling growth measurements were carried out after 7 days. Root length (cm) and shoot length (cm) were measured using a calibrated scale. Vigour index was determined by multiplying the germination percentage by seedling length (Equation 5).

(4) $$\text{Germination Percentage}=\frac{\left(\text{Number of Germinated Seeds}\right)\;}{\text{Total number of seeds}}\times 100$$

(5) Vigor Index=Germination Percentage×Seedling length

##### 2.7.5.2 Indole Acetic Acid (IAA) Production Assay

The 10‐day‐old bacterial culture was centrifuged to obtain cell‐free supernatants, and indole‐3‐acetic acid (IAA) production was assessed. For this colorimetric assay, 2 mL freshly prepared Salkowski reagent was combined with 1 mL of the supernatant. Salkowski reagent contained 0.5 M FeCl_₃_ in 35% perchloric acid (Gordon and Weber 1951). The mixture was then incubated at room temperature for 30 min in the dark to allow colour development. The presence of IAA was confirmed with the appearance of a pink to reddish colour (Patten and Glick 2002). Colour intensity was recorded through photography for documentation. For quantitative assessment, a standard curve was generated from 0 to 50 μg/mL using commercially available IAA diluted in sterile distilled water. Absorbances were taken at 530 nm using a UV–Visible spectrophotometer (Shimadzu UV‐1800). Estimation of the sample concentration was done based on the calibration curve (Patten and Glick 2002).

##### 2.7.5.3 Detection of Gibberellic Acid (GA)

Gibberellic acid (GA) detection was carried out using the 2,4‐dinitrophenylhydrazine (DNPH) colorimetric method. Briefly, both GA standard and sample solutions were taken in 1 mL quantities to a tightly stoppered Pyrex test tube. A control containing solvent without GA was also prepared. In each tube, 1 mL DNPH reagent was added and incubated at 30°C for 1 h, allowing formation of hydrazone. Then, 5 mL of 10% potassium hydroxide (KOH) in 80% methanol was added to each tube; the solution was mixed and allowed to stand for 5 min to develop colour. The appearance of a wine‐red colour indicated the presence of GA. The reaction mixture was then diluted with distilled water, and absorbance was measured at 430 nm and 540 nm using a spectrophotometer with the blank as reference. The measured absorbance values were assumed to be proportional to the GA standard concentration. The DNPH assay is based on the formation of coloured complexes between gibberellins and DNPH under alkaline conditions (Davies 2004).

### 2.8 Extraction of Semi‐Purified Biosurfactant

The cell‐free supernatant collected from the isolates was acidified by dropwise addition of 6 N HCl to a final pH of 2.0. The acidified supernatant was stored at 4°C overnight to induce biosurfactant precipitation. Followed by centrifugation at 10000 rpm for 20 min at 4°C. The supernatant was discarded, and the pellets were dissolved in alkaline distilled water (pH 8.0). As a solvent, Chloroform: Methanol (2:1) was added, followed by 20 min of vigorous shaking. The mixture was transferred to a separating funnel to allow phase separation, and the organic bottom layer was collected. These were transferred to a glass beaker and placed in a water bath under a fume hood at 45°C for 3 to 6 h, depending on evaporation ability, and were stirred occasionally.

### 2.9 Fourier Transform Infrared (FTIR) Spectroscopy

The biosurfactant extracts were lyophilized using a freeze‐dry system (Model: FreeZone2.5 by LabConco), and 2 mg of the purified, dried biosurfactant was thoroughly blended with 100 mg of spectroscopic‐grade dry KBr crystals. Followed by grinding into fine powder in an agate mortar and the homogeneous mixture was subsequently transferred into a standard die set, pressing under high mechanical pressure (130 MPa) to produce a thin, transparent pellet. The spectral data, for identifying the chemical functional groups of the biosurfactant, were scanned using an FTIR Spectrophotometer (Model: IRAffinity‐1S, Shimadzu Japan), ranging from wavenumbers 400 to 4500 cm^−1^. For each measurement, 25 scans were accumulated at a spectral resolution of 2 cm^−1^ using Happ‐Genzel apodization. A clean, blank KBr pellet was also scanned under the same conditions prior to sample execution, serving as the background reference.

### 2.10 Statistical Analysis

Descriptive statistics and initial data visualizations were performed using Microsoft Excel 365. Subsequent multivariate data analyses, ANOVA and post hoc tests, and advanced graphics were generated in RStudio using R version 4.1.1, with the packages ggplot2, pheatmap, prcomp, tidyverse, and Bioconductor.

## 3 Results

### 3.1 Hydrocarbon‐Utilizing Bacterial Isolates Found in Industrially Polluted Water

From the water sample collected from Tongi Turag Khal, contaminated with industrial waste, six bacterial cultures were isolated in nutrient agar medium enriched with diesel as a hydrocarbon source. Comparing the water using UV light with household‐use pond water as a negative control and diesel‐contaminated water as a positive control indicated the possible presence of hydrocarbons in the water sample (Figure S1). The water samples were found to be highly alkaline (pH > 8.4, total alkalinity 80–120 mg/L). Total hardness, that is, the total concentration of dissolved minerals (Ca^2+^ and Mg^2+^ ions) in water, was measured as 425 mg/L, which is considered very hard, along with ~180 mg/L measurement for carbonate. An overload of Nitrate (10 mg/L) was observed in the water sample. Except for copper (0.2 mg/L) and iron (1.0 mg/L), other elements like cyanuric acid, mercury, chlorine, bromine, chromium, lead, fluoride, and arsenic were in insignificant amounts or absent in the sample. The names IUBFASM‐tgbS1, IUBFASM‐tgbS2, IUBFASM‐tgbS3, IUBFASM‐tgbS4, IUBFASM‐tgbS5, and IUBFASM‐tgbS6 were assigned to the six cultures.

### 3.2 Isolated Bacterial Strains Showed Biosurfactant‐Producing Ability

Biosurfactant‐producing ability of the isolates was confirmed using the standard tests for assessing oil displacement, emulsification, and drop collapse activities. All isolates yielded positive results in the oil displacement assay; specifically, isolate IUBFASM_tgbS2 and IUBFASM_tgbS6 demonstrated the highest activity with a displacement area of 11.2 ± 0.35 cm^2^ and 10.34 ± 1.03 cm^2^, respectively, while IUBFASM_tgbS1 showed the lowest at 2.96 ± 0.37 cm^2^ (Figure 2b). However, when the extract was further purified using Chloroform: Methanol (2:1) solvent, the oil displacement ability showed a loss of efficiency but still showed the capacity for oil displacement, confirming the production of biosurfactant.

The emulsification index (E24%) of the six isolates in diesel and kerosene is illustrated in Figure 3c and Figure S2. IUBFASM_tgbS1, IUBFASM_tgbS2, and IUBFASM_tgbS6 showed high emulsification indices in both diesel and kerosene; however, variation was observed in the emulsification capacity of bacteria for diesel and kerosene, for example, IUBFASM_tgbS2 showed higher E24% in diesel but lower in kerosene.

All six bacterial cultures showed positive results from the drop collapse test, indicating the effectiveness of the generated surfactant to emulsify the oil. The emulsification index of the semi‐purified extract was lower than that of the cell‐free extract and is not reported here. IUBFASM_tgbS1 showed the highest drop collapse ability among all.

### 3.3 Biochemical and Molecular Characterization of the Bacterial Isolates

#### 3.3.1 Gram Staining Uncovers the Diversity of Bacterial Strains

The isolates (IUBFASM_tgbS1 and IUBFASM_tgbS6) were found to be rod‐shaped and Gram‐positive (+) according to Gram Staining, indicating the presence of a strong peptidoglycan layer on their cell walls. On the other hand, IUBFASM_tgbS2 and IUBFASM_tgbS5 were found to be Gram‐negative bacteria. IUBFASM_tgbS3 and IUBFASM_tgbS4 showed an ambiguous result in the Gram staining, which was reconfirmed using 3% KOH, but the ambiguity about the aforementioned cultures was not resolved.

#### 3.3.2 Metabolic and Enzymatic Activities Confirmed by Biochemical Tests

From the cumulative biochemical profiles and morphological properties depicted in Table 1, the six bacterial cultures can be categorized into four groups. Group 1 comprises exclusively IUBFASM_tgbS5, which is a Gram‐negative bacterium characterized by active glucose and lactose fermentation, gas production, indole synthesis, and a positive Methyl red reaction. The metabolic footprint is complemented by a complete absence of extracellular starch hydrolysis, negative citrate utilization, and negative oxidase reaction, which matches the profiling criteria of *Escherichia coli* .

**TABLE 1:** Biochemical test results of the six biosurfactant‐producing isolates.

Property	IUBFASM_tgbS1	IUBFASM_tgbS2	IUBFASM_tgbS3	IUBFASM_tgbS4	IUBFASM_tgbS5	IUBFASM_tgbS6
Plate ID*	S1i3	S1i4	S2	S3i5	S4	S3i4
Gram staining	+	−	+/−	+/−	−	+
Motility	+	+	+	+	+	+
Indole	−	−	−	−	+	−
Urease	−	−	−	−	−	−
Glucose fermentation	−	−	−	−	+	−
Lactose fermentation	−	−	−	−	+	−
Starch hydrolysis	+	+	−	+	−	+
Arabinose	+	−	−	++	+++	++
Citrate utilization	++	++	−	+	−	+
H_2_S production	−	−	−	−	−	−
Gas production	−	−	−	−	+	−
Oxidase	+	+	−	+	−	+
Catalase	+	+	+	+	+	+
MR	−	−	+	−	+	−
VP	+	−	−	+	−	+

* To match the sample numbers shown in the various figures, we have included the actual plate IDs from our practical work here.

The second group is comprised of gram‐positive environmental *Bacillus* isolates. IUBFASM_tgbS1 and IUBFASM_tgbS6 showed Gram‐positive staining. They showed starch hydrolysis capacity. Both IUBFASM_tgbS1 and IUBFASM_tgbS6 showed positive citrate utilization, positive oxidase activity, and positive Voges‐Proskauer (VP) reaction. Such specific fingerprint aligns with the *Bacillus subtilis* group characteristics. Both IUBFASM_tgbS1 and IUBFASM_tgbS6 also showed moderately positive results for L‐arabinose fermentation.

Group 3 comprises environmental strains Gram‐negative IUBFASM_tgbS2 and ambiguous IUBFASM_tgbS4. Both isolates showed positive oxidase activity, active citrate utilization, and extracellular amylase activity via starch hydrolysis. IUBFASM_tgbS4 demonstrated versatile metabolic routing and showed VP positive and moderate L‐arabinose fermentation.

The final cluster, group 4 is IUBFASM_tgbS3. Unlike other strains, it was negative for starch hydrolysis, negative for citrate utilization, and oxidase activity. It has a moderately positive MR reaction.

#### 3.3.3 Profiling Through 16S rRNA Sequencing

For correctly identifying the bacterial strain at the molecular level, 16S rRNA analysis was carried out. Based on the BLAST analysis with the NCBI 16S rRNA database, it was found that the microbes are from the *Bacillus, Stutzerimonas, Acinetobacter*, and *Enterobacteriaceae* genera (Table 2). To resolve the ambiguity about IUBFASM_tgbS3 and IUBFASM_tgbS4, the result was further confirmed using 16S rRNA‐based amplicon sequencing (Figure S3). For IUBFASM_tgbS3, the amplicon sequencing revealed the presence of multiple bacteria from *Bacillus* and *Acinetobacter*, showing properties from both. Due to the ambiguous morphological nature of IUBFASM_tgbS3 and IUBFASM_tgbS4, despite attempts at making pure culture, the presence of mixed culture was evident; hence, they will be referred to as consortium in the rest of the article.

**TABLE 2:** Molecular identification results of the isolates using 16S rRNA sequencing.

ID of the isolates	Practical plate ID*	Genus from taxonomy	BLAST alignment match	Percent identity	Bit score
IUBFASM_tgbS1	S1i3	*Bacillus*	*Bacillus spizizenii* strain NRRL	99.356	845
			*Bacillus subtilis* strain DSM 10	99.356	845
			*Bacillus inaquosorum* strain BGSC 3A28	99.356	845
			*Bacillus tequilensis* strain 10b	99.356	845
IUBFASM_tgbS2	S1i4	*Stutzerimonas*	*Stutzerimonas stutzeri strain DSM 5190*	100	859
IUBFASM_tgbS3	S2	*Bacillus*	*Bacillus cereus* strain FDAARGOS_1084	100	861
			*Bacillus mycoides* strain JAS06	100	861
		*Acinetobacter*	*Acinetobacter baumannii* ATCC17978	99.785	856
IUBFASM_tgbS4	S3i5	*Bacillus*	*Bacillus spizizenii* strain NRRL	100	841
			*Bacillus subtilis* strain DSM 10	100	841
			*Bacillus inaquosorum* strain BGSC 3A28	100	841
			*Bacillus tequilensis* strain 10b	100	841
IUBFASM_tgbS5	S4	*Enterobacteriaceae*	*Escherichia coli strain RHB34‐CO2*	100	859
			*Escherichia/Shigella* sp. *Strain krb19‐163*	99.785	854
IUBFASM_tgbS6	S3i4	*Bacillus*	*Bacillus spizizenii* strain NRRL	100	861
			*Bacillus subtilis* strain DSM 10	100	861
			*Bacillus inaquosorum* strain BGSC 3A28	100	861
			*Bacillus tequilensis* strain 10b	100	861

* To match the sample numbers shown in the various figures, we have included the actual plate IDs from our practical work here.

#### 3.3.4 Biofilm Formation Ability of the Isolates

The isolate's biofilm activity was evaluated by using a microtiter plate assay. The IUBFASM_tgbS3 isolate had the highest absorbance, followed by IUBFASM_tgbS1 and IUBFASM_tgbS6, indicating robust biofilm formation. IUBFASM_tgbS5 showed slightly lower biofilm formation compared to the other isolates. The biofilm formation pattern was similar among the isolates and consortia but was slightly reduced when the assay was performed after 10 days of incubation, allowing production of biosurfactants (Figure 4).

**FIGURE 4:**
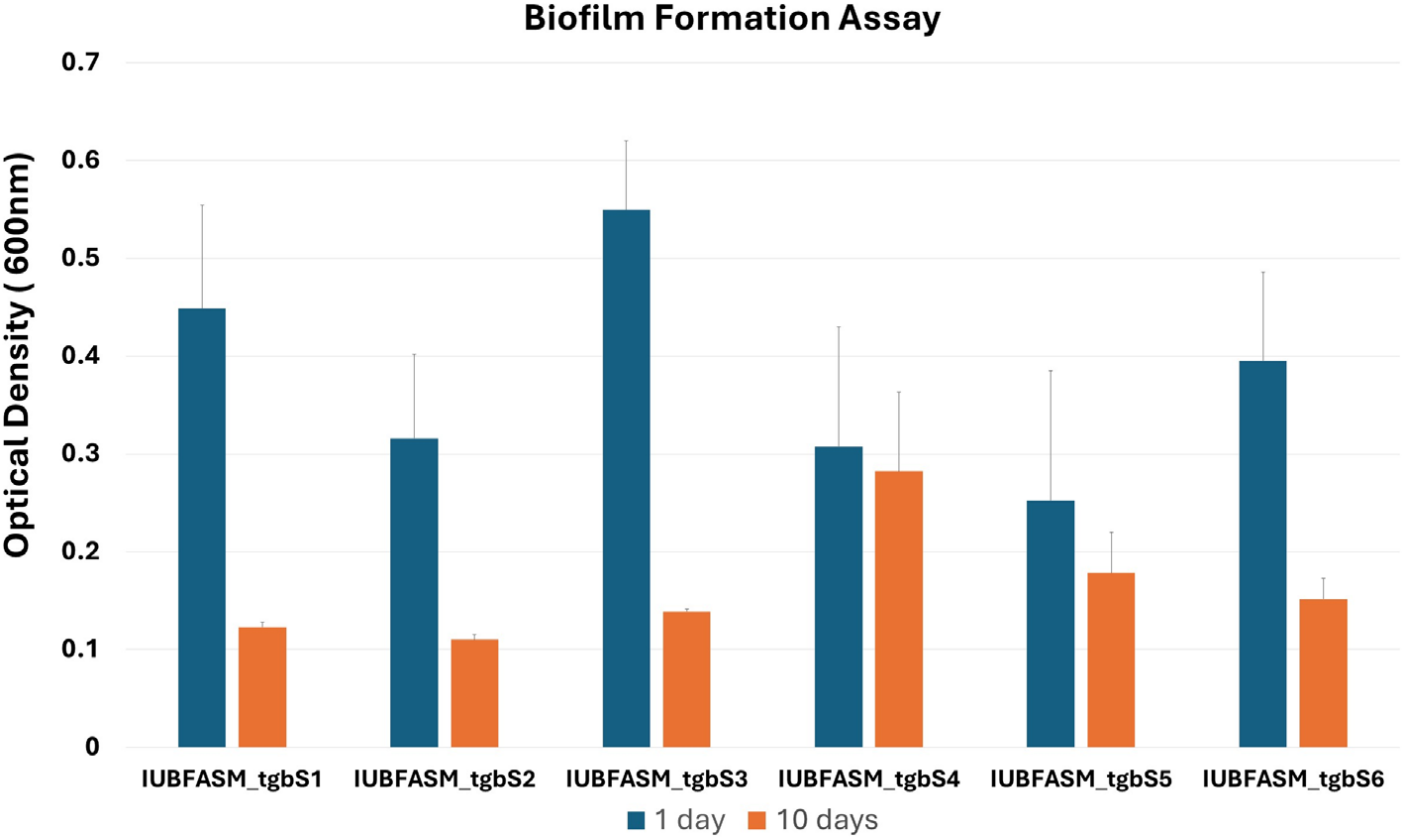
Biofilm formation ability of the isolates and consortia.

### 3.4 Characterization of the Surfactant Compound

#### 3.4.1 CTAB Agar Test Reveals Ionic Nature of the Biosurfactant

In the case of the CTAB method, a sign of ion matching between the cationic CTAB‐methylene blue agar complex and anionic biosurfactant was the formation of a dark blue halo surrounding the culture. IUBFASM_tgbS1 and IUBFASM_tgbS2 gave positive results, showing a dark blue halo (Figure 5). The other four isolates, however, showed moderate indication of blue colour, but the lower intensity indicates the small yield of the biosurfactants in these bacteria.

**FIGURE 5:**
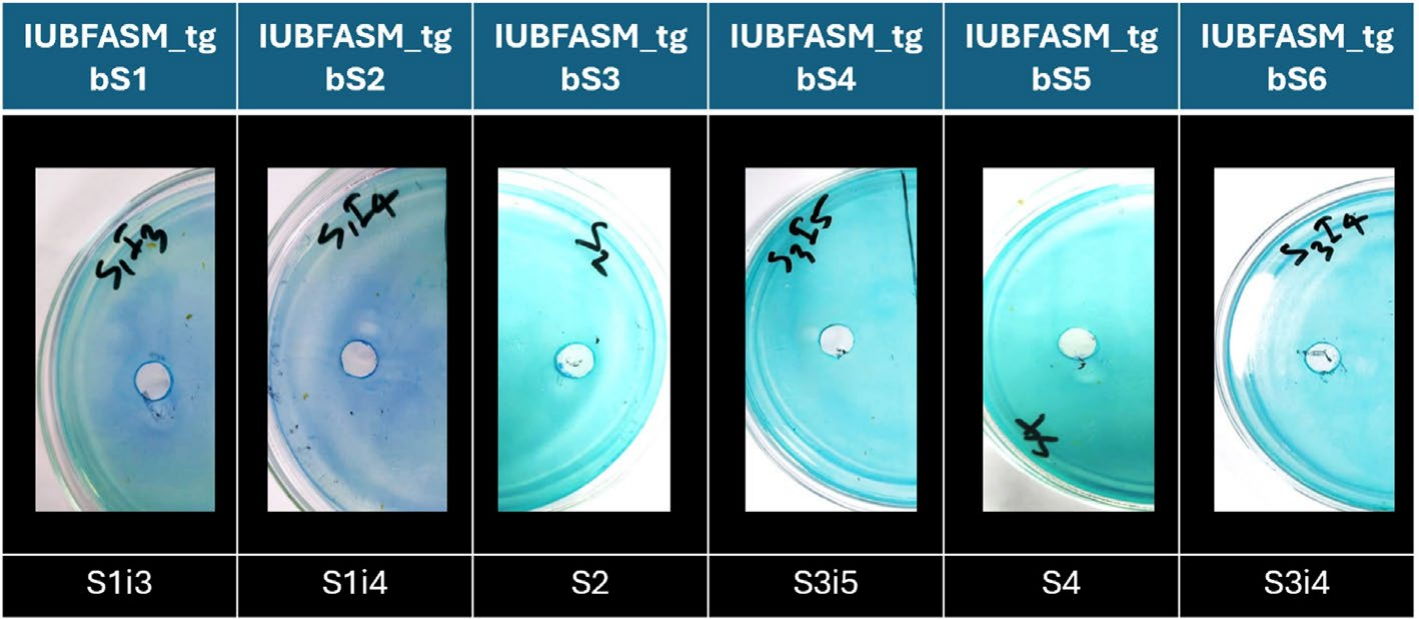
Biosurfactants showed a positive result at CTAB Methylene Blue agar complex.

#### 3.4.2 Impact of pH, Temperature, and Salinity on Emulsification

The emulsion was found to be stable over a temperature range from 40°C to 100°C. For IUBFASM_tgbS2 and IUBFASM_tgbS6, the emulsion index increased from 15% and 8% at 40°C to 18.18% and 13% at 80°C, respectively. At 100°C, IUBFASM_tgbS2 showed the highest stability of 44.44% emulsification index. Except for IUBFASM_tgbS4, all isolates and consortia showed increased emulsification index at higher temperatures (Figure 6).

**FIGURE 6:**
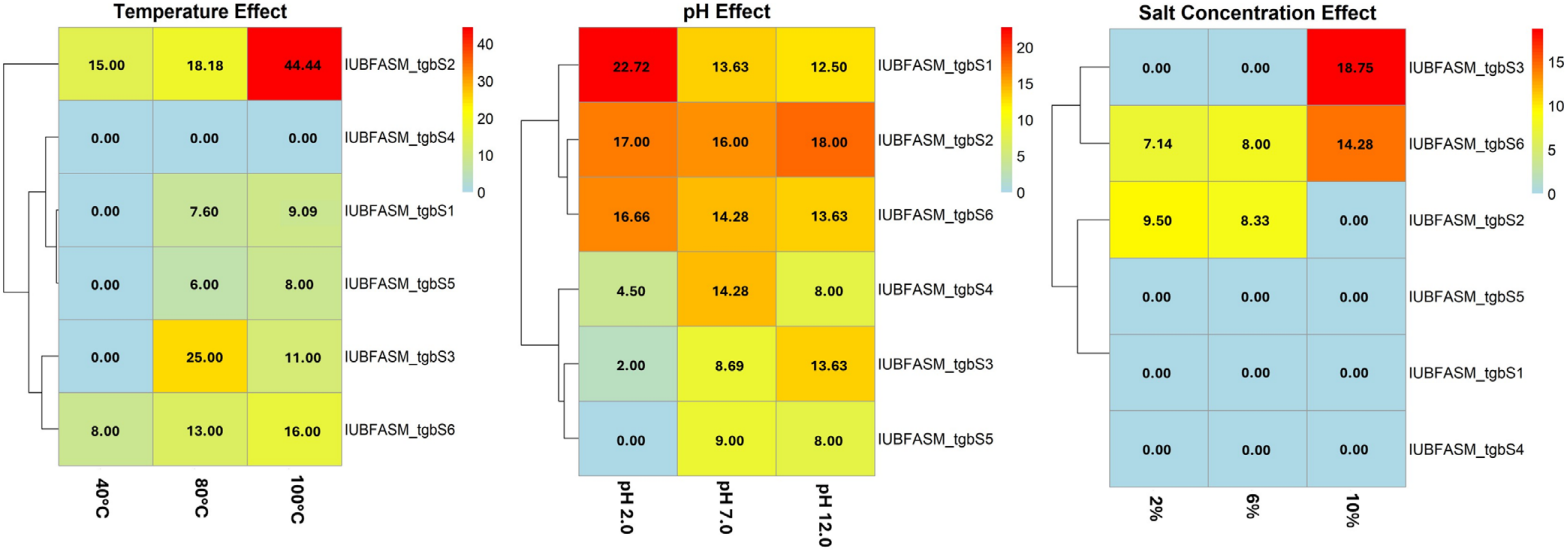
Emulsification Stability test showing the effect of temperature, salt, and pH on the emulsification index (E24%) of the isolates and consortia.

For all isolates, emulsion stability was lowest under acidic conditions, except for IUBFASM_tgbS1 and IUBFASM_tgbS6. IUBFASM_tgbS1, IUBFASM_tgbS2, and IUBFASM_tgbS6 showed stable emulsification index in both acidic and alkaline environments. The emulsion was found to be reasonably stable with the emulsion index in neutral conditions, although IUBFASM_tgbS1, IUBFASM_tgbS3, and IUBFASM_tgbS4 produced no results.

When the isolates were tested for emulsification index under 2%, 6%, and 10% saline conditions, IUBFASM_tgbS6 showed tolerance to salt and exhibited a higher emulsification index at high NaCl salt conditions. IUBFASM_tgbS2 showed moderate E24% in 2% and 6% salt but not under 10%. IUBFASM_tgbS3 showed good E24% at 10% salt concentration, but not at the lower salt concentration.

Overall, higher temperature 80°C–100°C and alkaline conditions (high pH) were found to be favourable for the isolates to generate higher emulsification. Among the six isolates and consortia, IUBFASM_tgbS6 showed the best stability of emulsification index across all pH, temperature, and saline conditions.

#### 3.4.3 Thin Layer Chromatography and FTIR Spectroscopy Identified the Lipopeptidic Nature of the Surfactants

Upon spraying the TLC plate with ninhydrin, all isolates except IUBFASM_tgbS2 and IUBFASM_tgbS5 displayed a pink colour, indicating that the biosurfactant contained peptide moieties. In TLC plates, different Rf values were noted, as shown in Table 3.

**TABLE 3:** The Retention factor (Rf) values of specific isolates and consortia.

Sample	*Rf* value	Possible compound*
IUBFASM_tgbS1	0.3	Iturin A or bacillomycin
IUBFASM_tgbS2	—	Non‐reactive to ninhydrin
IUBFASM_tgbS3	0.34	Iturin
IUBFASM_tgbS4	0.26	Fengycin
IUBFASM_tgbS5	—	Non‐reactive to ninhydrin
IUBFASM_tgbS6	0.52	Surfactin

* The possibility of the presence of these compounds is hypothetical based on tentative Rf values which is subjected to vary depending on chamber saturation, ambient humidity, plate activtion and slight solvent variation. The identitity of the compound was not assessed with help of standard purified compounds (Surfactin/fengycin/iturin/baciloomycin).

The surfactant compounds from the mixed cultures were semi‐purified and analysed by FTIR (Fourier Transform Infrared) spectroscopy, which showed peaks indicating their combined nature. The FTIR peaks of IUBFASM_tgbS3 in Table S1 show amide I and amide II linkages at 1647.24 and 1554.65 cm^−1^. It shows the lipid chain with a set of sharp peaks at 2960.78, 2925.10, and 2854.70 cm^−1^, corresponding to —CH stretching of the aliphatic beta‐amino fatty acid chains. There is also a peak at 3447.8 (broad) indicating —OH stretch that can originate from a rhamnose ring or glycolipid.

For IUBFASM_tgbS4, FTIR result shows a weak and broad peak at 3447.8 cm^−1^, indicating —OH stretch in rhamnose ring along with aliphatic C—H vibration, C—O of alcohol group, and C—O C ether group, indicating a mixed nature.

### 3.5 Additional Properties of the Bacterial Strains

#### 3.5.1 BATH Assay Measuring the Adherence Property of the Surfactant

The BATH (Bacterial Adhesion to Hydrocarbons) test was used to determine the % adherence of the isolates, which assesses cell hydrophobicity. Among the isolates, IUBFASM_tgbS4 showed the highest adherence in the BATH assay, followed by IUBFASM_tgbS2 (Figure 7). Lowest adherence was observed in IUBFASM_tgbS5, aligned with its other properties of biosurfactant production assays discussed above.

**FIGURE 7:**
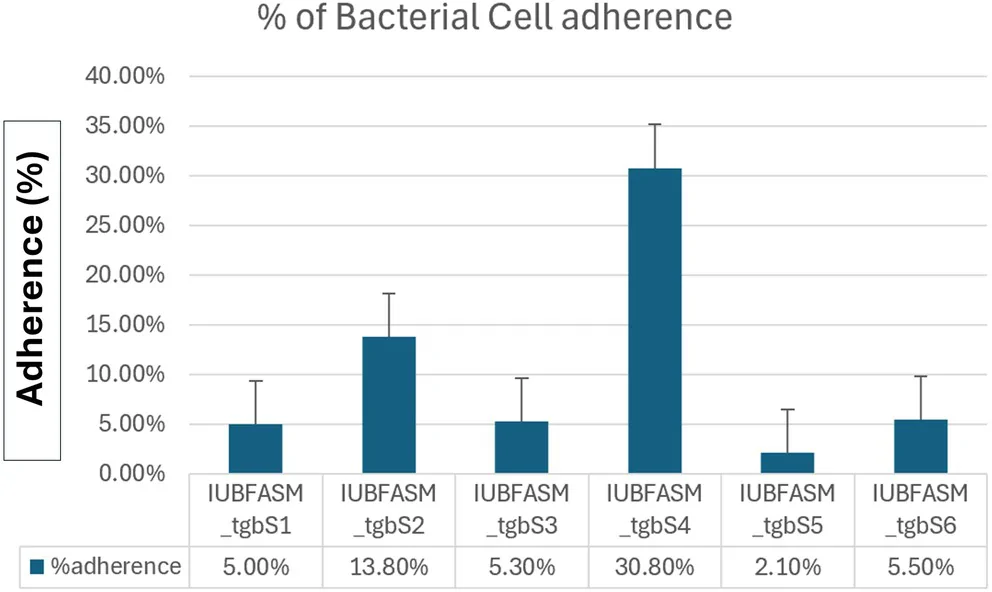
BATH assay.

#### 3.5.2 Agar Diffusion Assay Demonstrated Antibacterial Effect

In case of antibacterial activity, all the isolates show positive antibacterial activity against *Escherichia coli, Staphylococcus aureus, Vibrio cholerae Ogawa, Klebsiella*, and *Shigella* (Figure S4). Only IUBFASM_tgbS1 showed a negative result against *Staphylococcus aureus* .

#### 3.5.3 Antimicrobial Assays Verify Broad Bacterial Susceptibility

The isolates and consortia showed susceptible nature towards commercially available antibiotic discs of ampicillin, chloramphenicol, oxytetracycline, and azithromycin, confirming their safety profile in future applications. Only IUBFASM_tgbS3 showed a resistant profile against ampicillin and azithromycin.

#### 3.5.4 Isolates Showed Antifungal Activity Against Plant Pathogenic Fungus

IUBFASM_tgbS1, IUBFASM_tgbS2, IUBFASM_tgbS4, and IUBFASM_tgbS6 demonstrated antifungal activity in the well‐diffusion assay against plant pathogenic fungus *Colletotrichum*, characterized by axenic inhibition zones free of satellite colonies or fungal encroachment. Membrane‐filtered and unfiltered cell‐free extracts were compared to assess the efficiency of the extracts. IUBFASM_tgbS3 showed less activity for the non‐filtered one, and IUBFASM_tgbS5 showed the opposite trend, where the non‐filtered one gave good results. Except for filtered IUBFASM_tgbS5, IUBFASM_tgbS6, and non‐filtered IUBFASM_tgbS3, all isolates showed antifungal activity against *Colletotrichum* (Figure 8).

**FIGURE 8:**
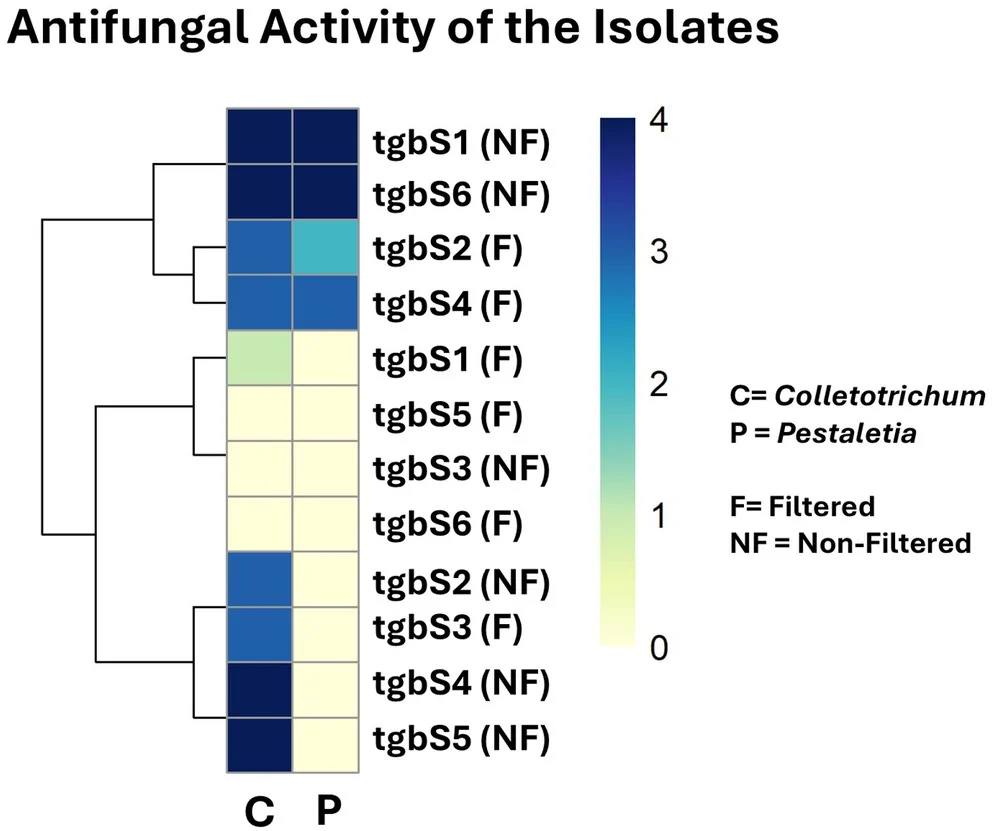
Antifungal activity against plant pathogenic fungus.

Reduced activity was observed against *Pestalotia*. Non‐filtered IUBFASM_tgbS1 and IUBFASM_tgbS6 showed continued high inhibition. Filtered IUBFASM_tgbS2 and IUBFASM_tgbS4 showed moderate activity. No activity was observed for IUBFASM_tgbS3 and IUBFASM_tgbS5. Overall, all filtered extracts showed inhibition against *Colletotrichum* except IUBFASM_tgbS5, whereas IUBFASM_tgbS2 and IUBFASM_tgbS4 filtered extracts showed inhibition against *Pestalotia*.

#### 3.5.5 Plant Growth Promotion Ability

Enhanced percentage of germination was observed in the *Oryza sativa* (indica) rice variety BRRI dhan28 upon application of the biosurfactant‐producing bacteria. Even upon application of 150 mM salt along with the bacteria, IUBFASM_tgbS1, IUBFASM_tgbS2, and IUBFASM_tgbS6 showed better germination. Highest germination was observed upon application of IUBFASM_tgbS4 and IUBFASM_tgbS3, followed by IUBFASM_tgbS5, without salt. IUBFASM_tgbS1 performed best to alleviate salt stress (Figure 9).

**FIGURE 9:**
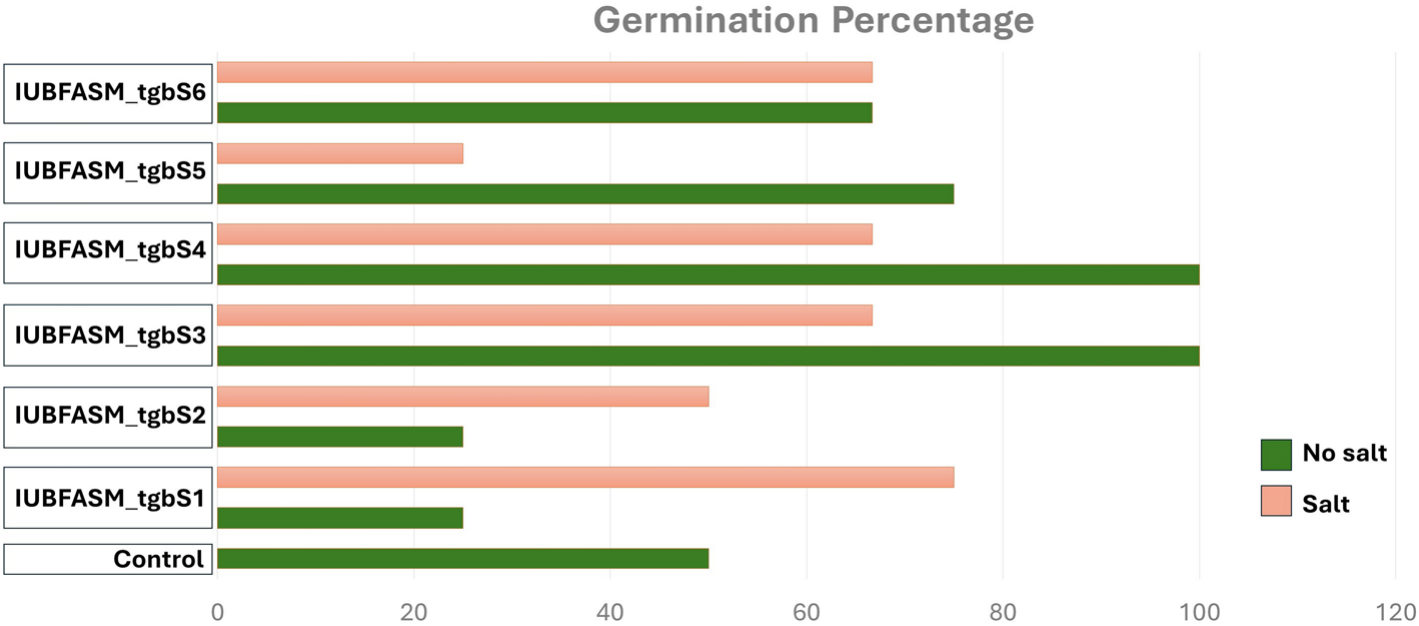
Germination percentage of BRRI dhan28 seeds upon application of a combination of biosurfactant‐producing bacteria and salt.

Growth promotion ability under salt stress was observed in all isolates except IUBFASM_tgbS6 (Figure 10), though it showed better ability during germination. Tolerance to salinity was observed after 3 days. IUBFASM_tgbS3 and IUBFASM_tgbs4 showed high growth promotion after 18 days, as shown in Figure S5.

**FIGURE 10:**
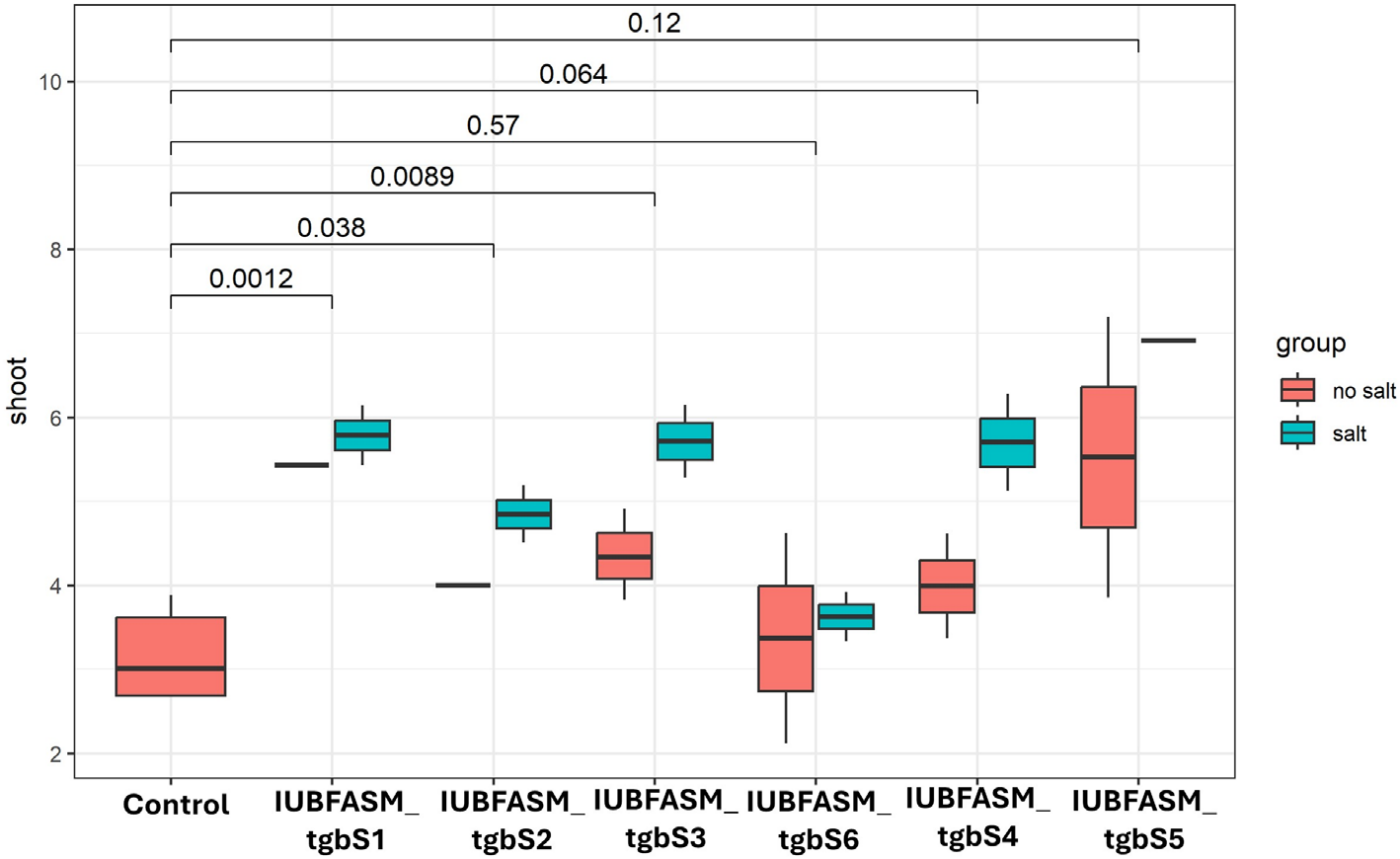
Plant growth promotion ability of the isolates after 3 days of 150 mM salt stress during the vegetative stage of the plant, showing shoot growth differences. The numbers above the box plots indicate the *p*‐value of the ANOVA post hoc test, showing the significance level of the difference between plants inoculated with bacterial culture and control plants without any bacterial inoculation.

IAA test confirmed the production of Indole acetic acid by IUBFASM_tgbS2 and IUBFASM_tgbS3, and strong IAA production by IUBFASM_tgbS5 (Table 4). Gibberellic acid production was confirmed in all isolates, with IUBFASM_tgbS2 and IUBFASM_tgbS4 exhibiting the highest levels, followed by IUBFASM_tgbS6, IUBFASM_tgbS3, and IUBFASM_tgbS5. Increased GA can help tolerate stress.

**TABLE 4:** Plant growth‐promoting hormone production by the isolates.

	IUBFASM_tgbS1	IUBFASM_tgbS2	IUBFASM_tgbS3	IUBFASM_tgbS4	IUBFASM_tgbS5	IUBFASM_tgbS6
IAA	−	+	+	−	++	−
GA	+	+++	++	+++	++	++

Considering the germination percentage, seedling growth on a petri dish for 18 days, both with salt stress and without salt, aligns with the IAA and GA production ability of the isolates and the consortiums. It was confirmed that IUBFASM_tgbS3 and IUBFASM_tgbS4 exhibited strong growth‐promoting ability.

### 3.6 Combined Functional Trait Analysis Revealed Specific Properties of the Isolates and Consortia

Principal component analysis was conducted to evaluate differences in the functions of the isolates and consortia across physiological traits. Total variance accounted for 68.9% of the cumulative variance (PC1: 44.0%, PC2: 24.9%) (Figure 11). We can see here that PC1 effectively segregated single isolates from multi‐strain consortia based on plant growth promotion and cell adherence activities (PGPR, Germ, BATH, and Biofilm), which showed positively along PC1.

**FIGURE 11:**
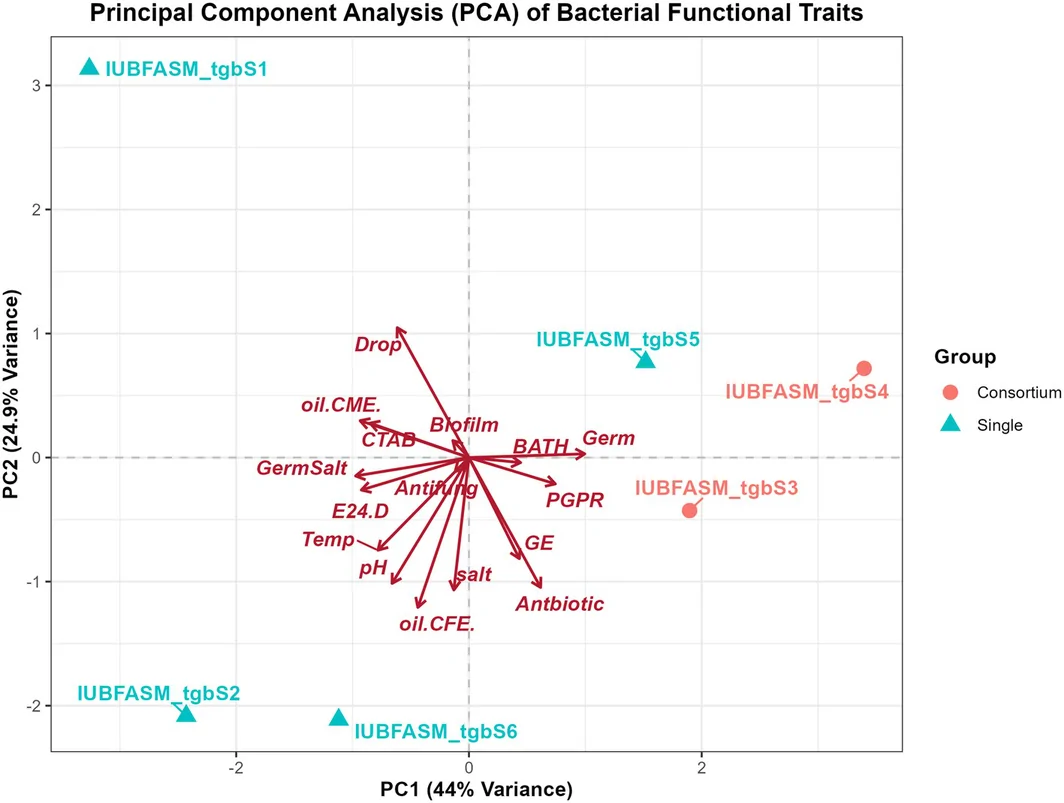
PCA biplot showing functional trait divergence of single bacterial isolates and consortia (here the cyan triangles represent individual isolates, red circles denote multi‐strain consortia, and red vectors indicate functional trait loadings. BATH, Bacterial Adherence to Hydrocarbons; Drop, drop collapse assay; E24.D, emulsification index in diesel; Germ, seed germination percentage; oil CME, oil displacement by purified extract; oil. CFE, cell free extract oil displacement; PGPR, plant growth promoting rhizobacteria traits).

Compared to most single isolates, the consortia IUBFASM_tgbS4 and IUBFASM_tgbS3 mapped prominently in the positive PC1 domain, aligning with adherence%, seed germination, and biofilm stability. On the other hand, individual strains exhibited biosurfactant production, for example, IUBFASM_tgbS1, as evidenced by the drop‐collapse assay and oil displacement of the purified compound. IUBFASM_tgbS2 and IUBFASM_tgbS6 showed associations with abiotic stress tolerance vectors such as temperature, extreme pH, and salt tolerance. IUBFASM_tgbS5 was the sole isolate that clustered near the consortia along PC1, indicating better PGPR activity. This clustering is illustrated in Figure S6 as a cluster heat map of the functional traits. Overall, the PCA biplot shows that the consortia help in coupling surface‐active biosurfactant production with surface adherence and plant growth promotion.

## 4 Discussion

The study reports the identification and isolation of six bacterial isolates and consortia with oil displacement property from industrial wastewater near Dhaka city, Bangladesh. These bacteria showed evidence of biosurfactant production in the oil displacement, drop collapse assay, and emulsification assay. Out of the six cultures, two are from the *Bacillus* genus, one from *Stutzerimonus*, and another is from the *Enterobacteriaceae* group. The other two bacterial cultures showed a mixed nature despite attempts to isolate a pure colony and were termed as consortia in the article. One mixed culture shared sequence identity with *Bacillus* and *Acinetobacter* genus, whereas another matched *Bacillus* and *Stutzerimonas* group. Their mixed nature was confirmed using genomic identifications as well as the nature of the produced surfactant. Identification of the bacterial consortia was confirmed through biochemical tests as well as Sanger and amplicon‐based 16S rRNA sequencing; however, ambiguity about some strains remained that cannot be overcome without multilocus typing that is beyond the scope of this report. The isolates and consortia also showed antimicrobial activity as well as plant growth promotion ability, making them viable for diverse applications as biocontrol agents and in plant growth promotion. Proper identification of the bacteria and the characterization of the surfactant produced by them are important for the utilization of these properties.

### 4.1 Identification of the Bacterial Genera

For the identification of bacteria, the common molecular level method, 16S rRNA gene sequencing, was used, which has 9 variable regions unique to different bacterial taxa. Using Sanger sequencing, all variable regions were covered to generate the sequence. Upon indication of mixed culture in two of the isolates, 16S rRNA‐based next‐generation amplicon sequencing was performed, targeting the variable V3 and V4 region for confirmation of the results. IUBFASM_tgbS1 showed the highest identity (99.35%) with 4 members of the *Bacillus subtilis* species complex, that is, *Bacillus spizizenii, B. subtilis, B. inaquosorum*, and *B. tequilensis* . IUBFASM_tgbS6 showed 100% similarity with this group. The isolates were able to grow in high pH conditions, characteristics relevant to *Bacillus spizizeni*i (AL Sharif et al. 2025). High sequence conservation within this complex precludes species‐level differentiation based solely on 16S rRNA sequencing and requires multilocus typing or whole‐genome sequencing. However, a follow‐up study on the genome analysis of this strain categorized it in the *Bacillus subtilis* group (Khatun and Elias 2025). The *Bacillus subtilis* group normally produces surfactin‐type biosurfactants, which are lipopeptides. In addition to the oil‐degrading ability, they show pharmacological effects like antibacterial, antifungal, anti‐mycoplasma, antiviral, anti‐thrombolytic, and anti‐inflammatory activities (Zhen et al. 2023). The *Bacillus* isolates discussed above consistently showed negative glucose fermentation in the carbohydrate fermentation tests, although *Bacillus* species typically show positive results in glucose metabolism. However, considering all other properties, this does not exclude them from being the *Bacillus subtilis* species complex, as they may vary among strains, and weak or delayed reactions have been reported.

IUBFASM_tgbS5 identity was confirmed as *E. coli* by their arabinose metabolism ability, along with the glucose and lactose fermentation capacity. It showed 100% similarity with *Escherichia coli* from the *Enterobacteriaceae* group. It also showed 99.78% identity with *Shigella*, but this was ruled out by the arabinose test, which was positive and characteristic of *E. coli* rather than *Shigella*. This can be a good root colonizer and potential biofertilizer candidate, also supported by their ability to produce auxin and gibberellic acid as well as to promote plant growth. It showed evidence of limited biosurfactant production. This might help the bacteria colonize the root, forming a biofilm by reducing surface tension. It can also help nutrient solubilization and have antimicrobial activity. Chigede et al. (2024) reported the isolation of a bacterium with 98.29% 16S rRNA identity to an *E coli* strain, indicating that *E coli* can produce a limited amount of biosurfactant, as observed in the current study. Biosurfactant production can regulate adherence, biofilm formation, and environmental applications.

IUBFASM_tgbS2 showed 99.36% identity with *Stutzerimonas stutzeri* from the 16S rRNA result previously classified as *Pseudomonus stutzeri*. It shows high emulsification ability, exhibits strong surface attachment, and biofilm formation mediated by biosurfactant chelation and extracellular polymeric substances, as we also observed in the current study.

IUBFASM_tgbS3 showed 100% similarity with the *Bacillus cereus* strain FDAARGOS_1084 chromosome and 99.79% similarity with *Acinetobacter baumannii* . Taxonomic identification from the QIIME2 16S rRNA amplicon‐seq analysis also indicated the abundance of both bacteria in the culture (Figure S3). This highlights the importance and concerns regarding the difficulties in isolating pure cultures from environmental samples. However, it also created scopes of studying the beneficial properties of both bacteria and taking precautions about any harmful effects when they are in a mixture. It showed moderate to low emulsification ability but high biofilm formation ability and plant growth promotion during germination. On the other hand, *Acinetobacter* is an aerobic, non‐motile, gram‐negative bacterium found in soil, freshwater, marine environments, as well as wastewater systems. *Acinetobacter bumannii* has shown the capacity to produce extracellular biosurfactants, predominantly composed of glycolipids, lipopolysaccharides, and complex polymeric compounds (Rosenberg and Ron 1999; Desai and Banat 1997; Mukherjee et al. 2006). Usually, both *Bacillus cereus* and *Acinetobacter baumannii* show positive results in the citrate utilization test; however, in this experiment, it appeared slightly negative, which indicates some strains might utilize citrate weakly and can take more time, or there can be competition with other sources like glucose. Their TLC results and FTIR peaks also support the mixed nature of the compounds, indicating secretion from both bacteria. There are reports of coexistence of strains from *Bacillus* and *Acinetobacter* in the environment, which can work together towards the biodegradation of pollutants (Yin et al. 2020).

Co‐isolation of *Bacillus* and *Stutzerimonas* (previously classified in the *Pseudomonas* group) is reported in the literature, mentioning they settle into a stable coexistence (Lyng and Kovács 2023). Another study by Liu et al. (2026) showed the beneficial coexistence of *Bacilus* and *stutzerimonas stutzeri* strain A1501 where BRI3 regulated the interaction in the consortium by stimulating A1501 biofilm formation. IUBFASM_tgbS4 showed properties of a mixed culture of *Bacillus* and *Stutzerimonas*. In 16S rRNA analysis, it showed 100% match with *Bacillus subtilis* complex, but in whole genome work (Fareha and Elias, unpublished) it showed similarity with *Stutzerimonas stutzeri*. The chance of a biasness of high similarity index with *Bacillus* can not be omitted due to its high copy number 16S rRNA genes compared to the *Stutzerimonas* species (Tanaka et al. 2013; Lalucat et al. 2006). However, the dominant bacteria in the consortia are *Stutzerimonas* as identified from the whole genome. Their TLC results and FTIR peaks also support the mixed nature resembling peaks relevant to surfactants produced by both *Bacilus* and *Stutzerimonas*.

### 4.2 Dynamics of Biosurfactant Production, Emulsification Efficiency, and Temporal Biofilm Formation

We observed differences in indices of emulsification ability between diesel and kerosene. Emulsification (E24%) reflects the ability to form stable oil–water emulsions, which depend not only on the amount of biosurfactant but also on high‐molecular‐weight bioemulsifiers and EPS (Extracellular Polymeric Substances) components such as polysaccharides, proteins, and lipids. Hence, for the IUBFASM_tgbS1 strain, we observed a higher emulsification index than the others but a lower oil‐displacement ability. With the purified surfactants, a loss of efficiency was observed in the oil displacement test and emulsification indices, possibly due to the degradation of active surfactant compounds during purification.

After 10 days of incubation with sequential subcultures to allow biosurfactant production, a lower biofilm level was observed in the isolates and consortia than in the one‐day‐grown cultures. The interaction of high concentrations of biosurfactants with anti‐biofilm properties can hamper biofilm formation and depends on biosurfactant concentration, nutrient availability, and hydrodynamics, which can alter behaviour. IUBFASM_tgbS4 showed a similar level of biofilm formation after 7 days. This may indicate the inter‐species metabolic cross feeding and signalling cross‐talk during long‐term incubation and triggering biofilm formation, recently reported in a *Bacillus* and *Stutzerimonas* co‐isolate (Liu et al. 2026).

The Bacterial Adherence to Hydrocarbons (BATH) assay is used to determine cell surface hydrophobicity, which is often influenced by biosurfactant production. The principle is based on the ability of bacterial cells to adhere to hydrocarbons (e.g., hexadecane, toluene, xylene), which correlates with their surface hydrophobicity (Rosenberg et al. 1980). In early log phases, when biosurfactant production is low, high hydrocarbon adherence is expected; in the late or stationary phases, low hydrocarbon adherence is expected. High adherence indicates high affinity, and low adherence indicates high biosurfactant production; this property is regulated by surfactant production. IUBFASM_tgbs4 consortia showed consistent biofilm production even after 10 days of incubation. This structural persistence was supported by high adherence % in the BATH assay, confirming their strong surface affinity and biofilm‐forming capability. IUBFASM_tgbS2 also showed higher cell adherence than the other isolates, which aligns with the intrinsic surface hydrophobicity typical of *Stutzerimonas* species, a member of the above‐mentioned IUBFASM_tgbS4 consortia.

### 4.3 Biosurfactant‐Producing Isolates Exhibit Potential in Bioremediation, Plant Growth Promotion, and Efficiency as a Biocontrol Agent

The antifungal assay against 2 plant‐pathogenic fungi revealed a difference between filtered and non‐filtered cell‐free extracts in their ability to inhibit fungal mycelial growth. Though the purpose of filtering through membranes was to remove any remaining bacterial cells from the cell‐free supernatant to omit the effect of the live bacterial cells. However, we can't ignore the possibility of filtration of the high molecular weight biosurfactant compound during the process of filtration. The biosurfactant can disrupt microbial membranes through surface‐tension reduction and pore formation. Considering all properties, IUBFASM_tgbS1, IUBFASM_tgbS2, and IUBFASM_tgbS6 showed higher germination percentages under salt stress than under no‐salt conditions. They are good surfactant producers, and the effect of the surfactants to alleviate salt stress was observed. IUBFASM_tgbS3, IUBFASM_tgbS4, and IUBFASM_tgbS5 were found to be plant growth promoters; under salt stress, they showed good shoot growth (Figure 10), though the germination percentage compared to the no‐salt condition was not good (Figure 9). This indicates that microbes' interactions for plant growth promotion are more pronounced during the post‐germination vegetative stage compared to the initial seed germination phase. However, the strong biosurfactant producers (IUBFASM_tgbS1 and IUBFASM_tgbS2) showed good salt stress mitigation evidence in both germination and vegetative stages, underscoring the importance of biosurfactants in combating salt stress. Biosurfactants reduce surface tension and improve water filtration around plant roots, ensuring water availability. Moreover, anionic biosurfactants can bind Na^+^ and other toxic ions, mitigating ionic stress on plants. They can also help other plant growth‐promoting bacteria to adhere to the root surface and colonize the root by forming biofilm. However, IUBFASM_tgbS6, though a good biosurfactant producer, showed moderate germination efficiency and shoot growth under salt stress.

In addition to the strains' antimicrobial properties, their biosurfactant‐producing ability can be useful against antibiotic‐resistant bacteria. By coating surfaces, biosurfactants reduce microbial colonization and can accelerate wound healing. This property can also prevent device‐associated infection and improve drug delivery systems. Since the isolates were found to be susceptible to commonly used antibiotics, they have a safer profile for potential future use. Due to limited resources, surfactant products from all strains could not be extracted as a purified compound, but this creates opportunities for future translational research that could leverage these resources.

We started with the hypothesis that this waterbody near Turag khal, with a diverse range of waste‐disposal sites, might harbour biosurfactant‐producing bacteria that evolved and adapted to the niche. The chemical profile of the water sample, that is, high alkalinity, high carbonate, high nitrate as an electron acceptor, and absence of harmful heavy metals like lead and arsenic, indicates the fact that these microbes might be producing the compounds for their survival and own physiology, but also playing a role in the bioremediation of this wastewater, mitigating toxicity, Therefore, optimizing the industrial utility of these evolved bacterial strains without disrupting their native microenvironment is a promising avenue for sustainable bioremediation.

## 5 Conclusions

We have successfully isolated four bacterial isolates and two consortia exhibiting diverse levels of biosurfactant production from an industrial waste‐effluent river near Dhaka city using the oil‐enrichment strategy. Most of the isolates that demonstrated measurable oil displacement and emulsification abilities were identified as members of the *Bacillus subtilis* complex, while one isolate belonged to the *Stutzerimonas* group. Isolate belonging to the *Enterobacteriaceae* group showed comparatively lower biosurfactant production but exhibited better plant growth promotion ability. The two consortia, *Bacillus‐Acinetobacter* and *Bacillus‐Stutzerimonas*, exhibited unique beneficial properties. All isolates and consortia displayed more or less antibacterial and antifungal activity against plant‐pathogenic fungi. Combined functional analysis revealed that the beneficial effect of the mixed cultures helps in coupling surface‐active biosurfactant production with surface adherence and plant growth promotion. Identification and characterization of these hydrocarbon waste‐adapted strains highlight their potential for eco‐friendly applications in pollution mitigation. This study underscores the value of industrial waste‐rich environments as reservoirs of metabolically versatile microorganisms with significant potential for identifying novel bacterial strains and unique bioactive compounds that enable microbial adaptation to extreme environmental conditions.

## Author Contributions

**Ummey Fatema Tun Amina:** data curation, formal analysis, writing – original draft, investigation, methodology. **Maliha Mahzabin:** formal analysis, methodology, investigation. **Sk. Shahriar Bin Rasul:** investigation, validation. **Sabrina M. Elias:** conceptualization, visualization, supervision, writing – review and editing, project administration, funding acquisition, validation, software, resources, data curation.

## Funding

This work was supported by Ministry of Science and Technology, Government of the People's Republic of Bangladesh, SRG‐252355; Independent University, Bangladesh, 2021‐SELS‐05.

## Ethics Statement

The study does not include any human subjects as study material and was approved by the institutional review board (IRB) of Independent University, Bangladesh.

## Conflicts of Interest

The authors declare no conflicts of interest.

## Supporting information


**Figure S1:** Comparison of Turag River water with artificially mixed diesel water, and clean water to compare presence of hydrocarbon upon exposure of UV light.
**Figure S2:** Emulsification index (E24%) of the six isolates and consortia in diesel and kerosene, along with the positive control 10% SDS.
**Figure S3:** Taxonomic identification result of 16S rRNA‐based amplicon sequence of the bacterial consortia.
**Figure S4:** Agar Diffusion Assay showing antibacterial property of the isolates and consortia.
**Figure S5:** Increase in shoot over time upon application of the isolates and consortia.
**Figure S6:** Cluster heat map showing grouping of isolates and consortia based on functional traits.
**Table S1:** Fourier Transform Infrared spectroscopy results of 2 mixed cultures.

## Data Availability

The data that support the findings of this study are available on request from the corresponding author. The data are not publicly available due to privacy or ethical restrictions.

## References

[emi470440-bib-0001] AL Sharif, B. , M. M. Golchini , A. Soorni , and R. Mehrabi . 2025. “Genomic and Enzymatic Insights Into α‐Amylase‐Producing Bacillus Spizizenii Strains Isolated From Isfahan Province, Iran.” PLoS One 20: e0333668. 10.1371/journal.pone.0333668. PMC12714195 41411302

[emi470440-bib-0002] Banat, I. M. , A. Franzetti , I. Gandolfi , et al. 2010. “Microbial Biosurfactants Production, Applications and Future Potential.” Applied Microbiology and Biotechnology 87: 427–444. 10.1007/s00253-010-2589-0 20424836

[emi470440-bib-0003] Bolyen, E. , J. R. Rideout , M. R. Dillon , et al. 2019. “Reproducible, Interactive, Scalable and Extensible Microbiome Data Science Using QIIME 2.” Nature Biotechnology 37: 852–857. 10.1038/s41587-019-0209-9. PMC7015180 31341288

[emi470440-bib-0004] Burkholder, P. R. , A. W. Evans , I. Mcveigh , and H. K. Thornton . 1944. “Antibiotic Activity of Lichens.” Proceedings of the National Academy of Sciences of the United States of America 30: 250–255. 10.1073/pnas.30.9.250. PMC1078706 16588653

[emi470440-bib-0005] Carolin C, F. , P. Senthil Kumar , G. Mohanakrishna , R. V. Hemavathy , G. Rangasamy , and T. Aminabhavi . 2023. “Sustainable Production of Biosurfactants via Valorisation of Industrial Wastes as Alternate Feedstocks.” Chemosphere 312: 137326. 10.1016/j.chemosphere.2022.137326. 36410507

[emi470440-bib-0006] Chigede, N. , Z. Chikwambi , I. D. Mpofu , and J. Madzimure . 2024. “Isolation and Characterization of Biosurfactant‐Producing Microbes Isolated From the Gastrointestinal System of Broiler Birds Fed a Commercial Diet.” Animal Biotechnology 35: 2263771. 10.1080/10495398.2023.2263771 PMC12674367 37814822

[emi470440-bib-0007] Cooper, D. G. , and B. G. Goldenberg . 1987. “Surface‐Active Agents From Two Bacillus Species.” Applied and Environmental Microbiology 53: 224–229. 10.1128/aem.53.2.224-229.1987 PMC203641 16347271

[emi470440-bib-0008] Dagenais Roy, M. , and E. Déziel . 2025. “Microbial Primer: Biosurfactants—The ABCs of Microbial Surface‐Active Metabolites.” Microbiology 171: 1604. 10.1099/mic.0.001604. PMC12413298 40911039

[emi470440-bib-0009] Datta, M. , and I. Chattopadhyay . 2024. “Applications of Microbial Biosurfactants in Human Health and Environmental Sustainability: A Narrative Review.” Discovery Medicine 1: 160.

[emi470440-bib-0010] Davies, P. J. 2004. Plant Hormones: Biosynthesis, Signal Transduction, Action! Springer Science & Business Media.

[emi470440-bib-0011] Deleu, M. , and M. Paquot . 2004. “From Renewable Vegetables Resources to Microorganisms: New Trends in Surfactants.” Comptes Rendus Chimie 7: 641–646.

[emi470440-bib-0012] Desai, J. D. , and I. M. Banat . 1997. “Microbial Production of Surfactants and Their Commercial Potential.” Microbiology and Molecular Biology Reviews 61: 47–64. 10.1128/mmbr.61.1.47-64.1997 PMC232600 9106364

[emi470440-bib-0013] Dunlap, L. , and D. Beckmann . 1988. “Soluble Hydrocarbons Analysis From Kerosene/Diesel.” In Proceedings of the Conference on Petroleum Hydrocarbons and Organic Chemicals in Ground Water 1988: Prevention, Detection and Restoration. National Water Well Association.

[emi470440-bib-0014] Femi‐Ola, T. , O. Oluwole , T. Olowomofe , and H. Yakubu . 2015. “Isolation and Screening of Biosurfactant‐Producing Bacteria From Soil Contaminated With Domestic Waste Water.” British Journal of Environmental Sciences 3: 58–63.

[emi470440-bib-0015] Gordon, S. A. , and R. P. Weber . 1951. “Colorimetric Estimation of Indoleacetic Acid.” Plant Physiology 26: 192–195. 10.1104/pp.26.1.192. PMC437633 16654351

[emi470440-bib-0016] Gudiña, E. J. , V. Rocha , J. A. Teixeira , and L. R. Rodrigues . 2010. “Antimicrobial and Antiadhesive Properties of a Biosurfactant Isolated From *Lactobacillus paracasei* ssp. Paracasei A20.” Letters in Applied Microbiology 50: 419–424. 10.1111/j.1472-765X.2010.02818.x 20184670

[emi470440-bib-0017] Jain, D. , D. Collins‐Thompson , H. Lee , and J. Trevors . 1991. “A Drop‐Collapsing Test for Screening Surfactant‐Producing Microorganisms.” Journal of Microbiological Methods 13: 271–279.

[emi470440-bib-0018] Khatun, T. , and S. M. Elias . 2025. “Genome Assembly and Gene Identification of Biosurfactant‐Producing Bacteria for Environmental Bioremediation.” Briefings in Bioinformatics 26: i46. 10.1093/bib/bbaf631.079.

[emi470440-bib-0019] Klindworth, A. , E. Pruesse , T. Schweer , et al. 2012. “Evaluation of General 16S Ribosomal RNA Gene PCR Primers for Classical and Next‐Generation Sequencing‐Based Diversity Studies.” Nucleic Acids Research 41: e1. 10.1093/nar/gks808. PMC3592464 22933715

[emi470440-bib-0020] Lalucat, J. , A. Bennasar , R. Bosch , E. García‐Valdés , and N. J. Palleroni . 2006. “Biology of *Pseudomonas stutzeri* .” Microbiology and Molecular Biology Reviews 70: 510–547. 10.1128/mmbr.00047-05. PMC1489536 16760312

[emi470440-bib-0021] Liu, Y. , M. Zhu , G. Zou , et al. 2026. “A Bacterial Ally for Nitrogen‐Fixing Biofilm: Enhancing the Rhizosphere Colonization of Stutzerimonas Stutzeri A1501 With Surfactin‐Producing *Bacillus velezensis* BRI3.” Applied and Environmental Microbiology 92: e00498. 10.1128/aem.00498-26. PMC13274376 42214380

[emi470440-bib-0022] Lyng, M. , and Á. T. Kovács . 2023. “Frenemies of the Soil: Bacillus and Pseudomonas Interspecies Interactions.” Trends in Microbiology 31: 845–857. 10.1016/j.tim.2023.02.003. 36878770

[emi470440-bib-0023] Makkar, R. S. , and S. S. Cameotra . 1997. “Biosurfactant Production by a Thermophilic *Bacillus subtilis* Strain.” Journal of Industrial Microbiology and Biotechnology 18: 37–42. 10.1038/sj.jim.2900349.

[emi470440-bib-0024] Md, F. 2012. “Biosurfactant: Production and Application.” Journal of Petroleum & Environmental Biotechnology 3: 124.

[emi470440-bib-0025] Mohan, P. K. , G. Nakhla , and E. K. Yanful . 2006. “Biokinetics of Biodegradation of Surfactants Under Aerobic, Anoxic and Anaerobic Conditions.” Water Research 40: 533–540. 10.1016/j.watres.2005.11.030. 16405945

[emi470440-bib-0026] Moldes, A. B. , R. Paradelo , D. Rubinos , R. Devesa‐Rey , J. M. Cruz , and M. T. Barral . 2011. “Ex Situ Treatment of Hydrocarbon‐Contaminated Soil Using Biosurfactants From *Lactobacillus pentosus* .” Journal of Agricultural and Food Chemistry 59: 9443–9447. 10.1021/jf201807r 21797277

[emi470440-bib-0027] Morikawa, M. , Y. Hirata , and T. Imanaka . 2000. “A Study on the Structure–Function Relationship of Lipopeptide Biosurfactants.” Biochimica et Biophysica Acta 1488: 211–218. 10.1016/s1388-1981(00)00124-4 11082531

[emi470440-bib-0028] Mukherjee, S. , P. Das , and R. Sen . 2006. “Towards Commercial Production of Microbial Surfactants.” Trends in Biotechnology 24: 509–515. 10.1016/j.tibtech.2006.09.005 16997405

[emi470440-bib-0029] Mulligan, C. N. 2005. “Environmental Applications for Biosurfactants.” Environmental Pollution 133: 183–198. 10.1016/j.envpol.2004.06.009. 15519450

[emi470440-bib-0030] Muthusamy, K. , S. Gopalakrishnan , T. K. Ravi , and P. Sivachidambaram . 2008. “Biosurfactants: Properties, Commercial Production and Application.” Current Science 94: 736–747.

[emi470440-bib-0031] O'Toole, G. A. 2011. “Microtiter Dish Biofilm Formation Assay.” Journal of Visualized Experiments 47: e2437. 10.3791/2437. PMC3182663 21307833

[emi470440-bib-0032] Patten, C. L. , and B. R. Glick . 2002. “Role of *Pseudomonas putida* Indoleacetic Acid in Development of the Host Plant Root System.” Applied and Environmental Microbiology 68: 3795–3801. 10.1128/AEM.68.8.3795-3801.2002. PMC124051 12147474

[emi470440-bib-0033] Rabbani, M. L. , and S. Sarker . 2017. “Pollution Sources Assessment of Turag River, Bangladesh.” IOSR Journal of Mechanical and Civil Engineering 14: 84–91.

[emi470440-bib-0034] Rani, M. , J. T. Weadge , and S. Jabaji . 2020. “Isolation and Characterization of Biosurfactant‐Producing Bacteria From Oil Well Batteries With Antimicrobial Activities Against Food‐Borne and Plant Pathogens.” Frontiers in Microbiology 11: 64. 10.3389/fmicb.2020.00064 PMC7093026 32256455

[emi470440-bib-0035] Rosenberg, E. , and E. Z. Ron . 1999. “High‐ and Low‐Molecular‐Mass Microbial Surfactants.” Applied Microbiology and Biotechnology 52: 154–162. 10.1007/s002530051502 10499255

[emi470440-bib-0036] Rosenberg, M. , D. Gutnick , and E. Rosenberg . 1980. “Adherence of Bacteria to Hydrocarbons: A Simple Method for Measuring Cell‐Surface Hydrophobicity.” FEMS Microbiology Letters 9: 29–33.

[emi470440-bib-0037] Rostás, M. , and K. Blassmann . 2009. “Insects Had It First: Surfactants as a Defence Against Predators.” Proceedings of the Royal Society B: Biological Sciences 276: 633–638. 10.1098/rspb.2008.1281 PMC2660939 18986976

[emi470440-bib-0038] Saiyam, D. , A. Dubey , M. A. Malla , and A. Kumar . 2024. “Lipopeptides From Bacillus: Unveiling Biotechnological Prospects—Sources, Properties, and Diverse Applications.” Brazilian Journal of Microbiology 55: 281–295. 10.1007/s42770-023-01228-3 PMC10920585 38216798

[emi470440-bib-0039] Salihu, A. , I. Abdulkadir , and M. Almustapha . 2009. “An Investigation for Potential Development on Biosurfactants.” Biotechnology and Molecular Biology Reviews 3: 111–117.

[emi470440-bib-0040] Sharma, M. , and S. Kaur . 2015. “Inhibitory Potential of Lactobacillus Species Isolated Fromfermented Dairy Products Against Escherichia Coli and *Staphylococcus aureus* .” Journal of Gastrointestinal Infections 3: 45–50.

[emi470440-bib-0041] Simpson, D. R. , N. R. Natraj , M. J. Mcinerney , and K. E. Duncan . 2011. “Biosurfactant‐Producing Bacillus Are Present in Produced Brines From Oklahoma Oil Reservoirs With a Wide Range of Salinities.” Applied Microbiology and Biotechnology 91: 1083–1093. 10.1007/s00253-011-3326-z. 21562978

[emi470440-bib-0042] Singh, A. , J. D. Van Hamme , and O. P. Ward . 2007. “Surfactants in Microbiology and Biotechnology: Part 2. Application Aspects.” Biotechnology Advances 25: 99–121. 10.1016/j.biotechadv.2006.10.004 17156965

[emi470440-bib-0043] Tahzibi, A. , F. Kamal , and M. Mazaheri Assadi . 2004. “Improved Production of Rhamnolipids by a *Pseudomonas aeruginosa* Mutant.” Iranian Biomedical Journal 8, no. 1: 25–31.

[emi470440-bib-0044] Tanaka, Y. , H. Nanamiya , K. Yano , K. Kakugawa , F. Kawamura , and K. Ochi . 2013. “rRNA (Rrn) Operon‐Engineered *Bacillus subtilis* as a Feasible Test Organism for Antibiotic Discovery.” Antimicrobial Agents and Chemotherapy 57: 1948–1951. 10.1128/aac.02604-12. PMC3623351 23335737

[emi470440-bib-0045] Van Der Vegt, W. , H. C. Van Der Mei , J. Noordmans , and H. J. Busscher . 1991. “Assessment of Bacterial Biosurfactant Production Through Axisymmetric Drop Shape Analysis by Profile.” Applied Microbiology and Biotechnology 35: 766–770. 10.1007/bf00169892.

[emi470440-bib-0046] Xia, W. , Z. Du , Q. Cui , et al. 2014. “Biosurfactant Produced by Novel Pseudomonas sp. WJ6 With Biodegradation of n‐Alkanes and Polycyclic Aromatic Hydrocarbons.” Journal of Hazardous Materials 276: 489–498. 10.1016/j.jhazmat.2014.05.062. 24929788

[emi470440-bib-0047] Yin, C.‐F. , Y. Xu , and N.‐Y. Zhou . 2020. “Biodegradation of Polyethylene Mulching Films by a Co‐Culture of Acinetobacter sp. Strain NyZ450 and Bacillus sp. Strain NyZ451 Isolated From *Tenebrio molitor* Larvae.” International Biodeterioration & Biodegradation 155: 105089. 10.1016/j.ibiod.2020.105089.

[emi470440-bib-0048] Zhen, C. , X. F. Ge , Y. T. Lu , and W. Z. Liu . 2023. “Chemical Structure, Properties and Potential Applications of Surfactin, as Well as Advanced Strategies for Improving Its Microbial Production.” AIMS Microbiology 9: 195–217. 10.3934/microbiol.2023012. PMC10113169 37091822

