# Supplementary material for: Waste‐Contaminated Sites Near Dhaka City Harbour Specific Biosurfactant‐Producing Bacillus and Stutzerimonas Strains With Potential Biotechnological Applications
Source: Environ Microbiol Rep. 2026 Oct 23;18(5):e70440. doi: 10.1111/1758-2229.70440 (PMC13692813; doi:10.1111/1758-2229.70440)
Supplement: Supplementary file 1 [file EMI4-18-e70440-s001.pdf]

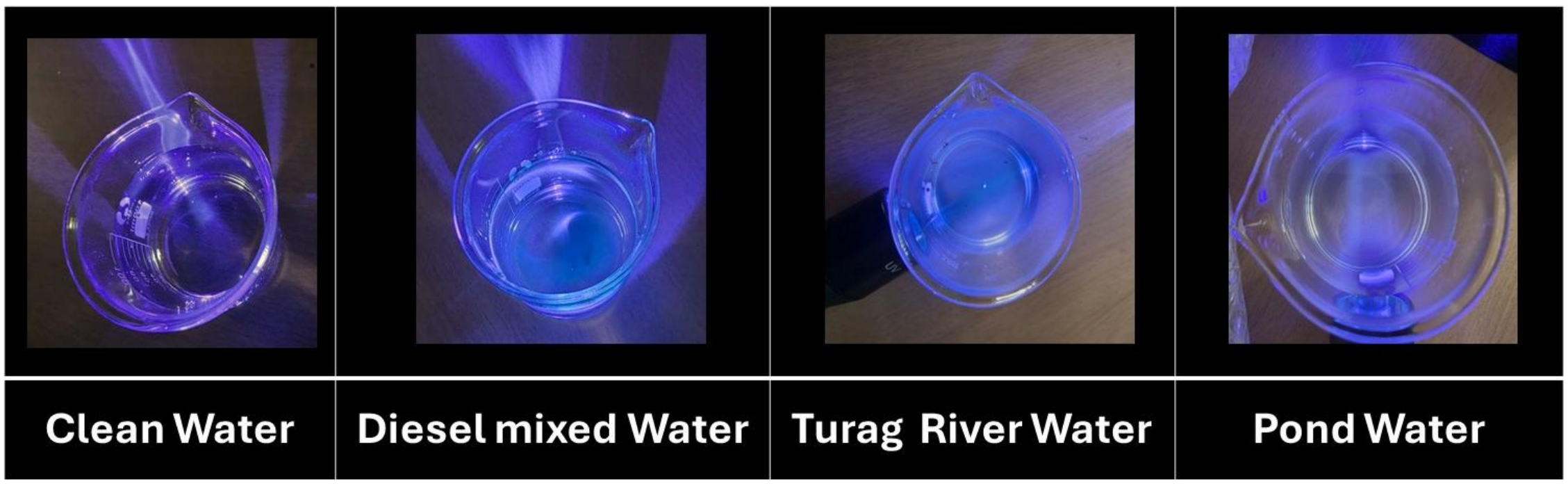

Supplementary Figure 1: Comparison of Turag River water with artificially mixed diesel water, and clean water to compare presence of hydrocarbon upon exposure of UV light

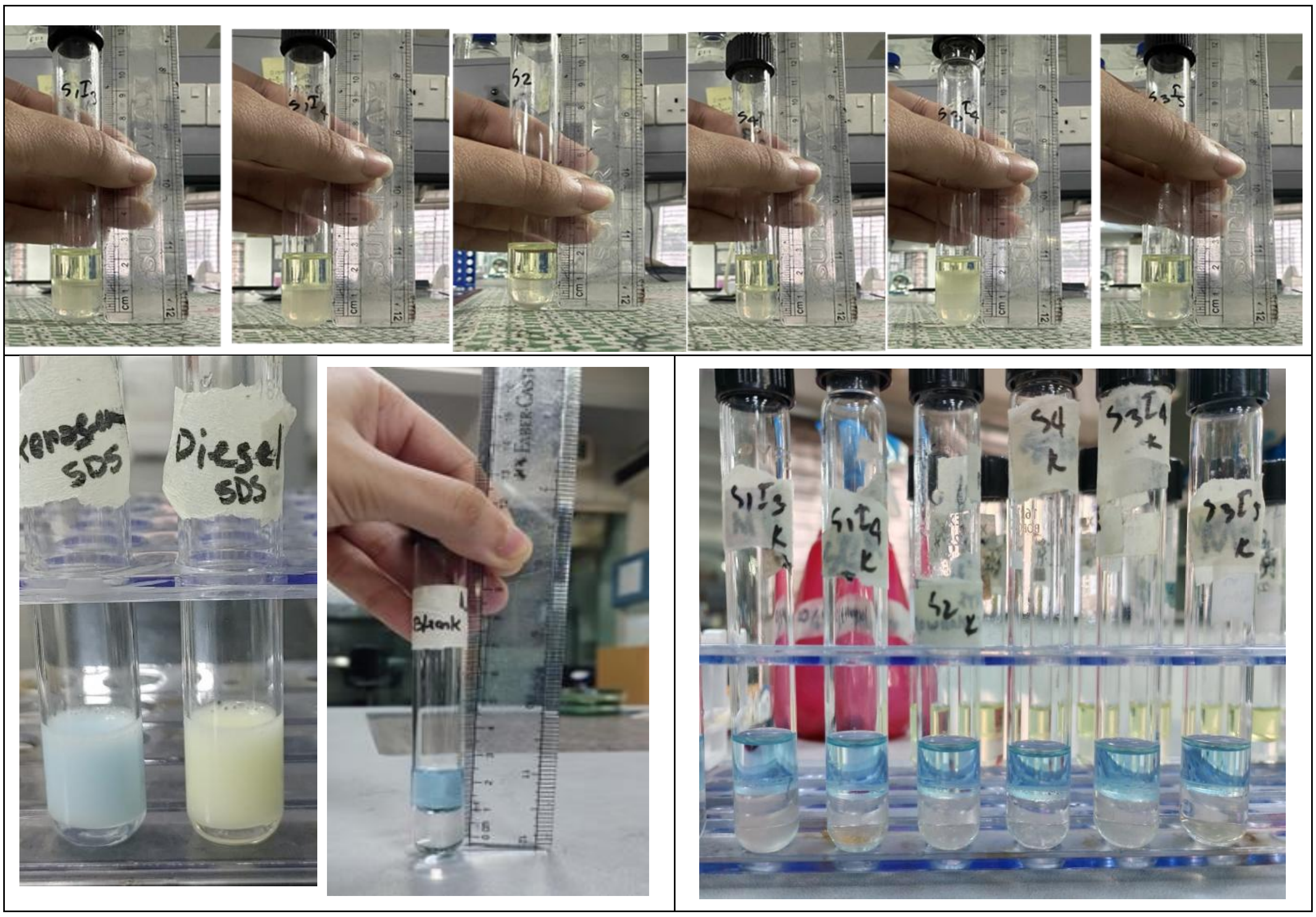

Supplementary Figure 2: Emulsification index (E24%) of the six isolates and consortia in diesel and kerosene, along with the positive control 10% SDS

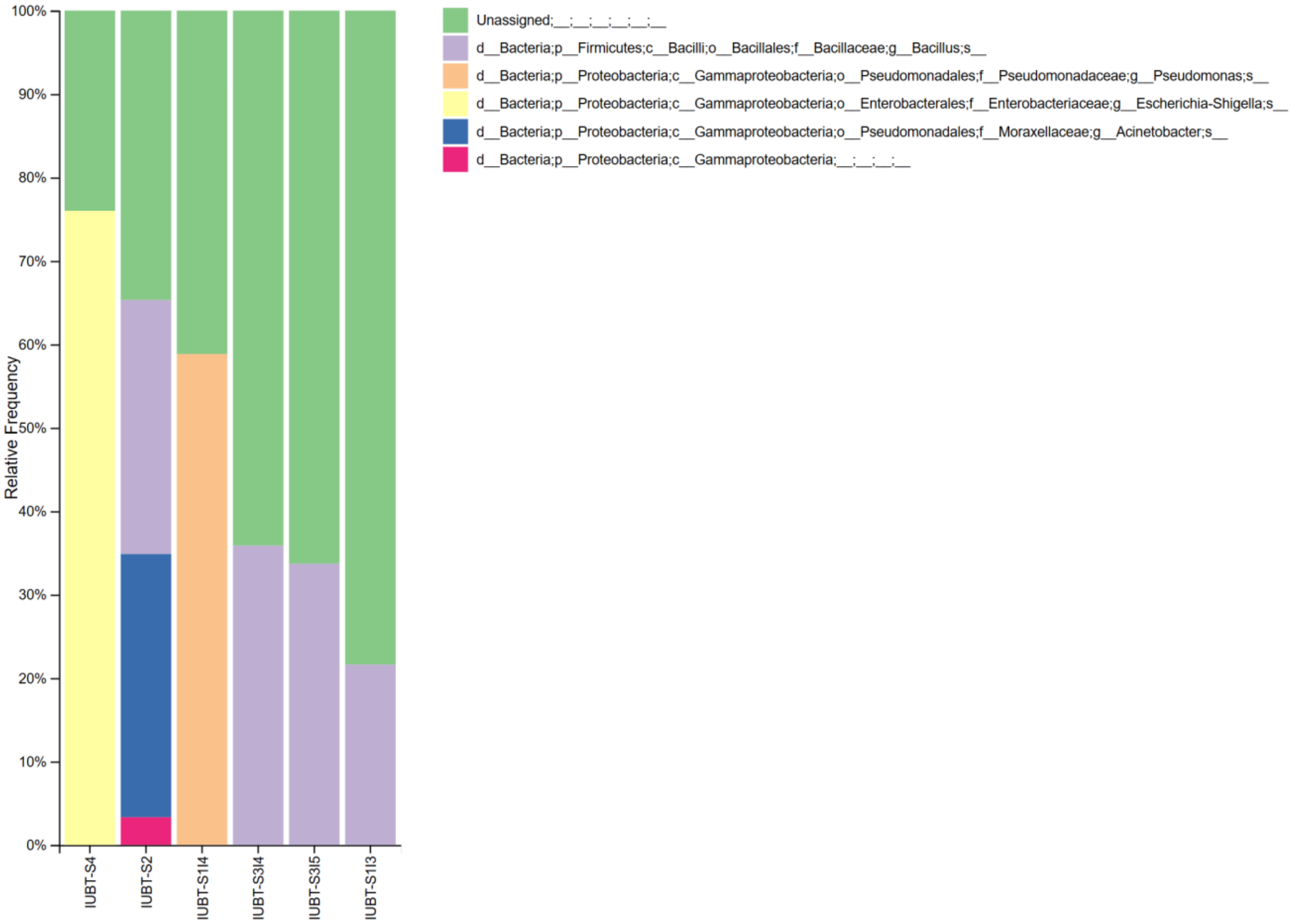

Supplementary Figure 3: Taxonomic identification result of 16S rRNA-based amplicon sequence of the bacterial consortia.

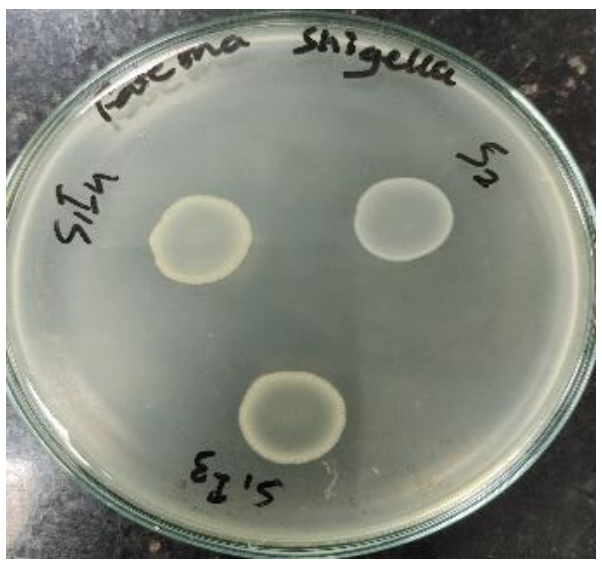

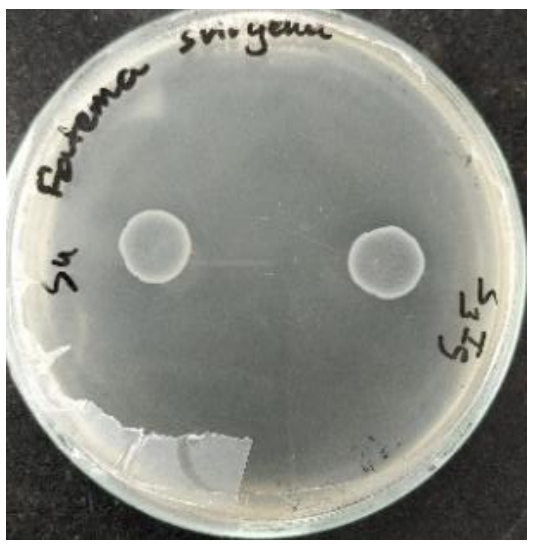

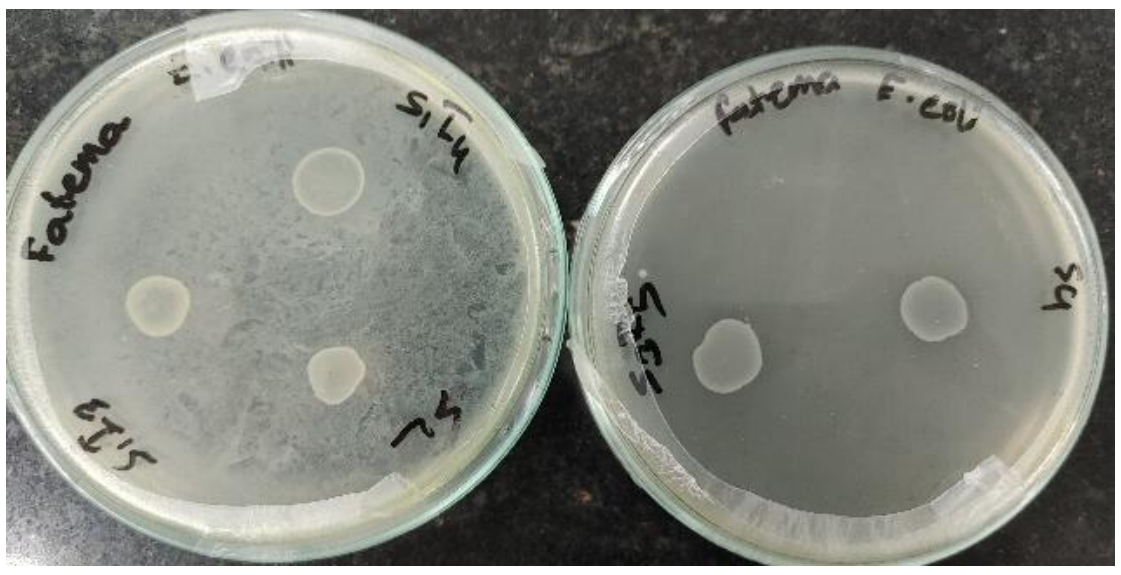

Against *Shigella*	Against *E coli*

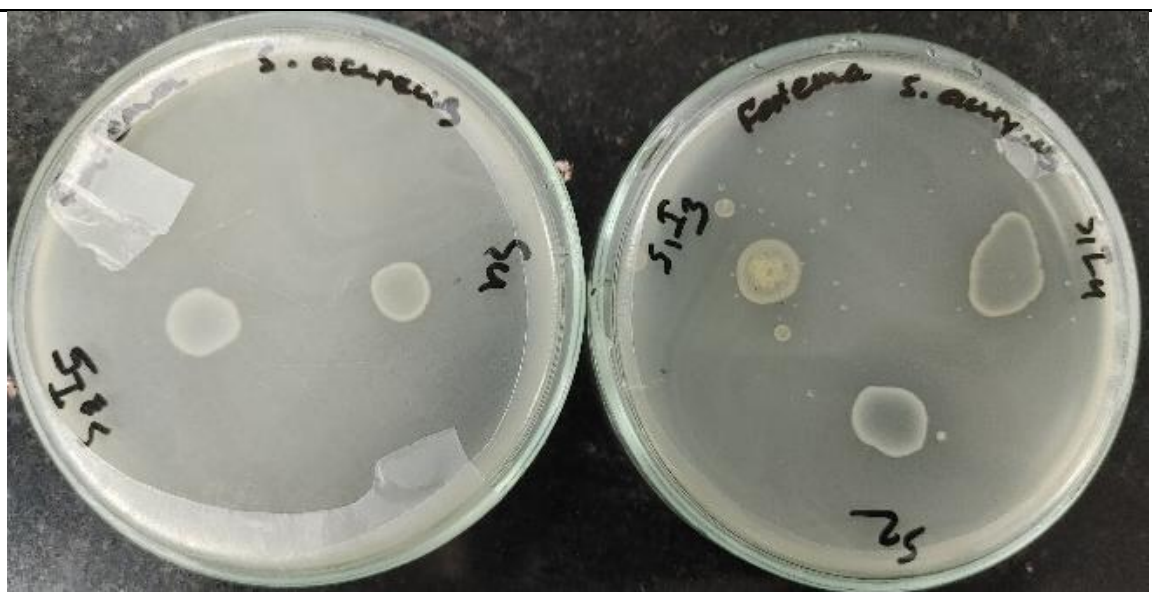

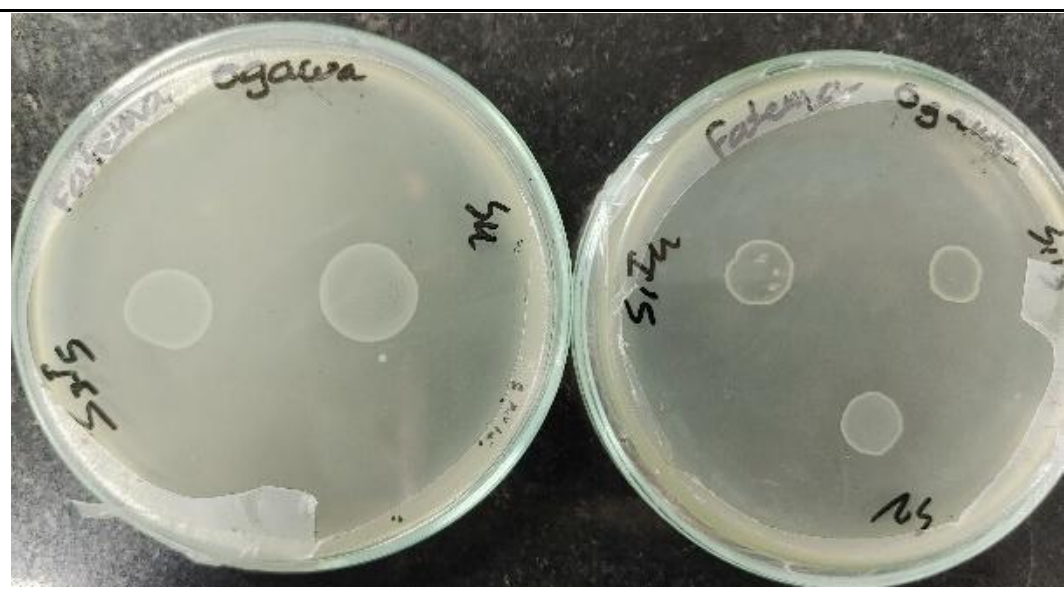

Against *S. Aureus*	Against *Ogawa*

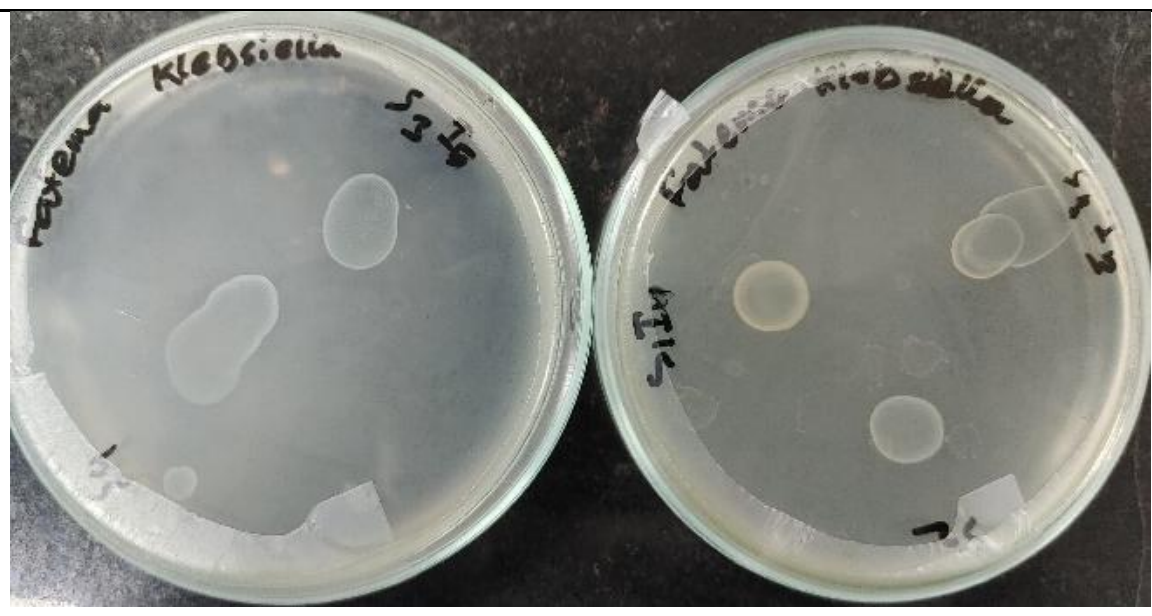

Against *Klebsiella*

Supplementary Figure 4: Agar Diffusion Assay showing antibacterial property of the isolates and consortia

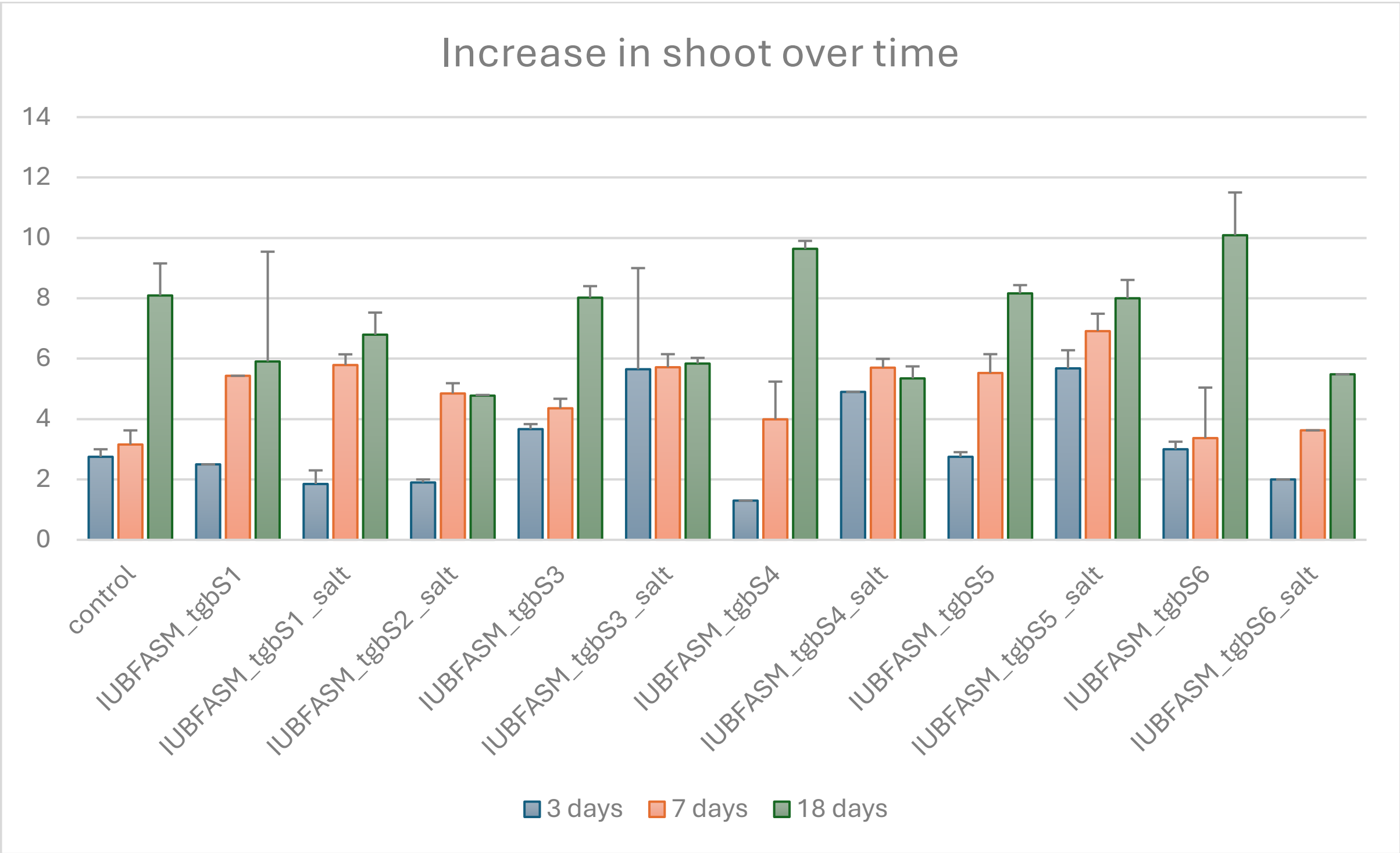

Supplementary Figure 5: Increase in shoot over time upon application of the isolates and consortia

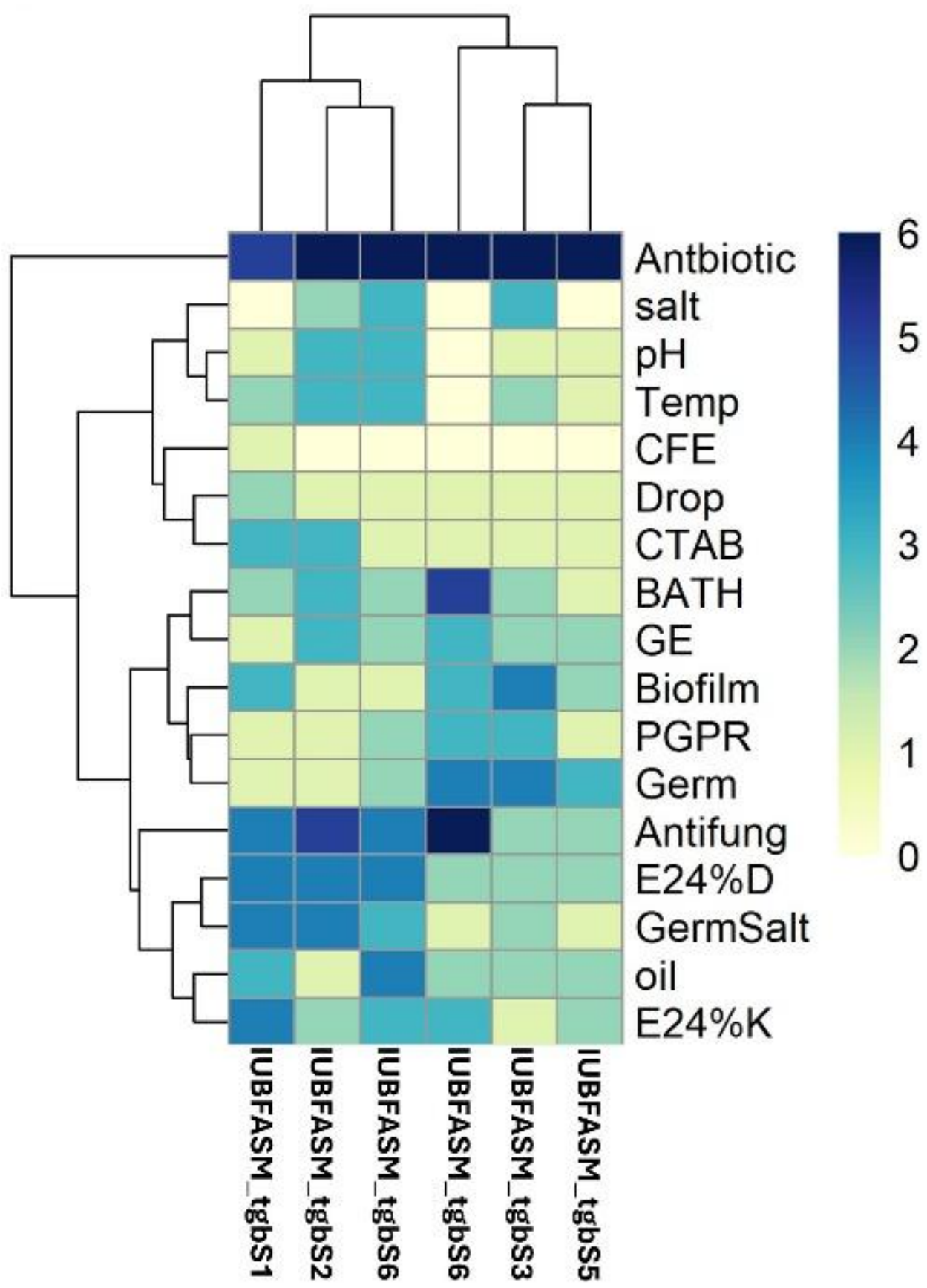

Supplementary Figure 6: Cluster heat map showing grouping of isolates and consortia based on functional traits.

Supplementary Table 1: Fourier Transform Infrared spectroscopy results of 2 mixed cultures

Functional Groups	Theoretical Range (cm^{-1})	Observable Peak (cm^{-1})	Comments
**IUBFASM_tgbS3**			
-OH or -NH stretch	3500-3200	3447.8 (broad)	-OH stretch or -NH stretch
C-H stretch	3000-2700	2850-2960	Aliphatic C-H vibration
C-O stretch	1075-1000	1024.2	C-O of alcohol group
-C-O-C-	1140-1070	1095.59	C-O-C ether group
C=O	1665-1780	1647.2 (weak)	
=C-O-H-	1320-1210	1261.5 (weak)	C-O stretch (carboxylic)
**IUBFASM_tgbS4**			
-OH stretch/-NH stretch	3500-3200 cm-1	3447.8 (weak and broad)	OH stretch in rhamnose possibly
-NH bending	>3000 (shoulder band)	Not observed	
C-N stretch	1230 -1030	Overlapped 1022-1098	Not significant
C-N bending	1620	Not observed	
C-H stretch	3000-2700	2850-2960	Aliphatic C-H vibration
C-O stretch	1075-1000	1022.29	C-O of alcohol group
-C-O-C-	1140-1070	1097.52	C-O C ether group
C=O	1665-1780	1743.68 (weak)	
